# Antigenic diversity and dengue disease risk

**DOI:** 10.21203/rs.3.rs-3214507/v1

**Published:** 2023-08-02

**Authors:** Lin Wang, Angkana T. Huang, Leah C. Katzelnick, Noémie Lefrancq, Ana Coello Escoto, Loréna Duret, Nayeem Chowdhury, Richard Jarman, Matthew A. Conte, Irina Maljkovic Berry, Stefan Fernandez, Chonticha Klungthong, Butsaya Thaisomboonsuk, Piyarat Suntarattiwong, Warunee Vandepitte, Stephen Whitehead, Simon Cauchemez, Derek A.T. Cummings, Henrik Salje

**Affiliations:** 1. Department of Genetics, University of Cambridge, Cambridge CB2 3EH, United Kingdom; 2. Laboratory of Infectious Diseases, National Institute of Allergy and Infectious Diseases, National Institutes of Health, Bethesda, MD 20892, USA; 3. Coalition for Epidemic Preparedness Initiative, Washington DC, USA; 4. Viral Diseases Branch, Walter Reed Army Institute of Research, Silver Spring, MD 20910, USA; 5. Department of Virology, Armed Forces Research Institute of Medical Sciences, Bangkok, Thailand; 6. Queen Sirikit National Institute of Child Health, Bangkok, Thailand; 7. Mathematical Modelling of Infectious Diseases Unit, Institut Pasteur, Université Paris Cité, CNRS UMR 2000, Paris, France; 8. Department of Biology and Emerging Pathogens Institute, University of Florida, Gainesville, FL 32611, USA

## Abstract

Many pathogens continuously change their protein structure in response to immune-driven selection, resulting in weakened protection. In addition, for some pathogens such as dengue virus, poorly targeted immunity is associated with increased risk of severe disease, through a mechanism known as antibody-dependent enhancement. However, it remains a mystery whether the antigenic distance between an individual’s first infection and subsequent exposures dictate disease risk, explaining the observed large-scale differences in dengue hospitalisations across years. Here we develop an inferential framework that combines detailed antigenic and genetic characterisation of viruses, and hospitalised cases from 21 years of surveillance in Bangkok, Thailand to identify the role of the antigenic profile of circulating viruses in determining disease risk. We find that the risk of hospitalisation depends on both the specific order of infecting serotypes and the antigenic distance between an individual’s primary and secondary infections, with risk maximised at intermediate antigenic distances. These findings suggest immune imprinting helps determine dengue disease risk, and provides a pathway to monitor the changing risk profile of populations and to quantifying risk profiles of candidate vaccines.

Antigenically variable pathogens present a major threat to human health. The ability of viruses, such as SARS-CoV-2 and influenza, to continuously change their genetic structure in response to the selective pressure of population immunity severely complicates control efforts^1–3^. In the case of dengue virus (DENV), an arbovirus that infects over 100 million people each year, the situation is even more complex^4,5^. Individuals with high DENV antibody titres are protected from infection and developing severe disease^6–8^. However, individuals with sub-neutralising antibody titres have been shown to have the highest risk of severe disease, through multiple hypothesised mechanisms including antibody-dependent enhancement^6,7,9–12^.

It has been shown that in an endemic setting, there are shifts in the antigenic properties of the DENV strains that circulate within any year, and long-term trends with circulating viruses tending to become more antigenically distant over decadal time frames^13^. However, it remains unknown if the antigenic relationship between circulating viruses and those that have previously infected individuals has any bearing on the risk of severe disease. Knowing this could improve our ability to predict the potential risk of newly circulating viruses. It would also provide biological insight into a role for antigenic imprinting to explain an individual’s long-term disease risk, where the specific virus that first infects an individual largely determines future disease risk when they are exposed to antigenically different viruses^14–16^. Further, the specific antigenic properties of strains used in vaccines could be evaluated for their potential to cause disease from generating sub-neutralisation titres, the magnitude of which may differ across settings due to heterogeneity in strain-specific immune histories and the antigenic characteristics of circulating viruses^17,18^.

Understanding the role of antigenic space in determining disease risk is highly complex. We need a detailed characterisation of the viruses circulating within a community over long time periods, the antigenic properties of the circulating viruses, and an understanding of who is getting sick in that same setting. Hospitalised cases in surveillance systems are also overwhelmingly from secondary infections, which means that the majority of primary infections and their antigenic signature are unobserved^19^. Here we overcome these hurdles through combining detailed genetic (N=2,587 viruses) and antigenic (N=348 viruses) characterisation of DENV isolated in Bangkok, Thailand, from 1994 to 2014, with long-term serotype and age-specific linelist case data from a large children’s hospital in the city (N=15,281 cases in individuals 1–14y) (Figure 1A, Figure S1, and Methods). We develop a mathematical framework that integrates over birth-cohorts lifetime exposures to the virus to explore whether the specific serotypes and antigenic distance between viruses causing primary and secondary infections are linked to disease risk.

## Antigenic and genetic characterisation of dengue viruses

**Figure 1.**
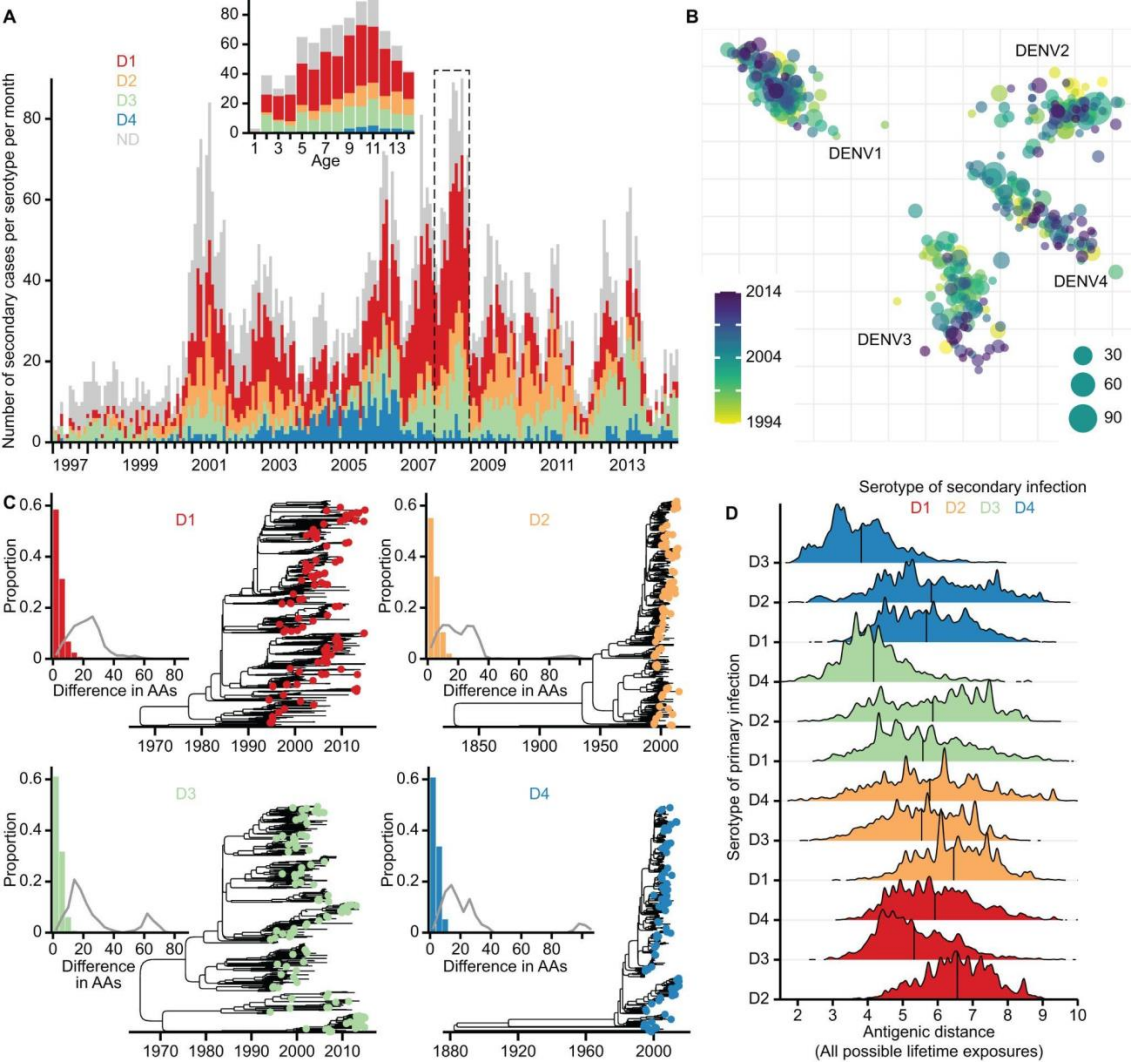
Long-term hospital-based dengue case data along with the antigenic and genetic characterisation of dengue viruses in Bangkok, Thailand. **(A)** Monthly number of secondary dengue cases hospitalised in the Queen Sirikit National Institute of Child Health in Bangkok, Thailand, from 1997 to 2014. Infecting age and year were known for each case, with 69.7% of cases diagnosed with the infecting serotype. The inset illustrates the aggregation of the original case linelist data into the serotype and age-specific case counts per year. Table S7 summarises the age groups analysed for each year. **(B)** Two-dimensional antigenic map of 2,594 Thailand viruses across four serotypes, coloured by year of isolation. Each coloured circle indicates one of the 348 dengue viruses antigenically characterised using PRNT assay. The size of each circle indicates the number of sequenced viruses placed onto the corresponding map location. Serotype clusters are labelled. Each grid square side in any direction represents one unit of antigenic distance, which is equivalent to a twofold dilution of antiserum in the PRNT assay. **(C)** Time-calibrated maximum clade credibility phylogenies built with sequenced viruses from each serotype. Coloured circles at the tips of each phylogeny indicate viruses selected for antigenic characterisation. In the inset, coloured bars indicate the distribution of the amino acid (AA) differences across the whole genome of each sequenced virus as compared to its genetically closest virus used for antigenic characterisation, while the grey curve indicates the same distribution but compares each sequenced virus to an antigenically characterised virus randomly selected from those of the same serotype. **(D)** Distribution of antigenic distances separating viruses that are possibly responsible for an individual’s primary and secondary infections, which is stratified by the serotypes of the possible primary and secondary infecting viruses (i.e., serotype pair) and coloured by the identity of the secondary serotype. Vertical line in each distribution indicates the corresponding mean antigenic distance. Figures S7, A to B, present the same distributions but use antigenic distance data derived from the original 3D antigenic map and the 3D antigenic map enhanced with sequenced viruses, respectively.

We use a detailed characterisation of antigenic space, where viruses from Thailand and 20 other countries were used in neutralisation assays^13^. The viruses were individually tested by plaque (immunofocus) reduction neutralisation test (PRNT), with antisera from African green monkeys that had been inoculated with reference viruses that capture the breadth of DENV antigenic diversity (total of 8,643 neutralisation measurements)^13^. This allowed us to build a three-dimensional antigenic map, where the Euclidean distance between any two viruses is inversely proportional to the capacity for the antiserum raised against one virus to neutralise the other virus. To increase the resolution of the population antigenic profile in any year, we used full genome sequences to place additional viruses^20^ circulating in Bangkok from 1994 to 2014 on the antigenic map (Figure 1B). For each sequenced virus, we identified the genetically closest virus that was used to construct the original antigenic map and gave the sequenced virus the same coordinates. As the original map was developed with a broad representativeness of circulating viruses, there was little genetic difference between those used to build the map and the extra sequenced viruses (median difference of 4 amino acids across the genome) (Figure 1C). We consider the sequenced viruses from a serotype as representative of the viruses of that serotype circulating in Bangkok in the study period. This enhanced antigenic map therefore can be used to capture the changing antigenic profile of the virus population.

By considering the viruses that are possibly responsible for an individual’s primary and then secondary infections (i.e., sequential in time), we find that the overall mean inter-serotype distance is 5.74 units, ranging from a mean distance of 3.82 units for individuals with a primary DENV-3 infection followed by a DENV-4 infection (equivalent to a 14.1 fold change in dilution), to a mean distance of 6.46 units for individuals with DENV-1 followed by a DENV-2 infection (equivalent to an 88.0 fold change in dilution) (Figure 1D, Table S3). The antigenic distance can be significantly greater or shorter depending on the specific viruses within each serotype responsible for the primary and secondary infections (coefficient of variation of 0.24 across the different serotype pairs) (Table S3).

## Risk of severe dengue by serotype pair and antigenic distance

We use the age and serotype information of cases caused by secondary infections that attended our surveillance hospital to explore whether the specific order of serotypes an individual was infected with, as well as the antigenic distance between the specific viruses, is linked to risk of disease. Secondary infections were identified as part of standard protocols in this hospital based on IgM/IgG ratios between acute and convalescent samples^19^. While we do not know the serotype, virus, and age of each individual’s primary infection, we can use the age and serotype information along with our observed distribution of viruses in prior years to integrate over all possible infection histories, where we also estimate the annual force of infection by serotype (Methods).

To model the sequential infections for each case, we assume that the force of infection for a given serotype is equivalent for people at risk of primary and secondary infection, but following a primary infection, susceptibility to other serotypes is mediated by cross-protection that lasts one year, with the magnitude of cross-protection estimated by our model. We assume no homotypic reinfections^10,11^. To assess the role of antigenic diversity in determining disease risk from a secondary infection, we develop three different models where the probability of becoming a case in hospital depends on (1) the identity of the secondary infecting serotype only, (2) the serotype of both the primary and secondary infection, and (3) the serotype of the primary and secondary infection and the antigenic distance between the two infecting viruses (full model). These different model hypotheses were compared using several model comparison metrics (Tables S1, S2). The model that considers the serotype of both the primary and secondary infection performed better than the model only considering the serotype of the secondary infection (ΔDIC=63). The full model that further incorporates the antigenic distances separating viruses has the best performance as compared to the model only considering the serotype of both the primary and secondary infection (ΔDIC=32). This provides strong evidence that the serotypes of both the historic primary infecting virus and the secondary virus are needed to explain the observed patterns of disease in hospitals, and moderate evidence that the specific viruses of both infections are also important.

**Figure 2.**
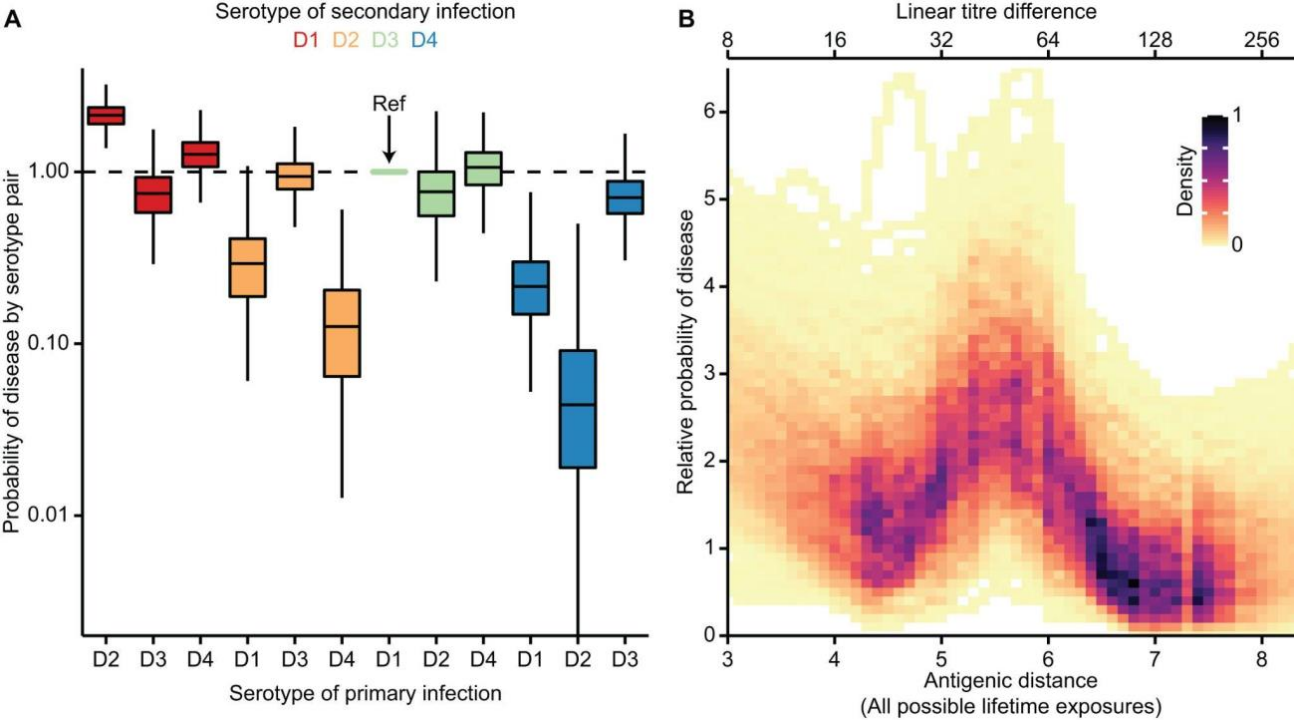
Probability of disease following a secondary infection. **(A)** Probability of disease by serotype pair. Primary DENV-1 followed by secondary DENV-3 serves as the reference for comparison. In each boxplot, the central horizontal line, edges of box, and whiskers indicate the median, interquartile range (IQR), and 1.5 * IQR of the posterior distribution, respectively. Colour indicates the identity of the secondary serotype. **(B)** Relationship between the relative probability of disease and the antigenic distance between the two viruses that are responsible for an individual’s primary and secondary infections. Colour indicates the density of the data points in the posterior estimates, using all antigenic distance data for 13,170,100 pairwise potential primary and secondary infecting viruses. Darker colour indicates higher density.

In the full model, we find that the probability of disease was greatest for those with a primary DENV-2 and a secondary DENV-1 infection, with a relative risk of 2.15 (95% credible interval (CrI): 1.49–2.85) compared to a DENV-1 followed by DENV-3 infection (the reference pair) (Figure 2A). Disease risk was consistently lower for secondary DENV-4 infections, consistent with other findings^21^. We estimate that in the first year following primary infection there is a 69.8% (95% CrI: 61.1–76.7%) reduction in the probability of infection, consistent with temporary cross-protection as identified elsewhere^22^. The mean antigenic distance between each serotype pair was not associated with the probability of disease for that pair (p-value 0.77). We find similar relative risk of disease by serotype-pair for models that did and did not include antigenic distances between virus pairs (Figure S2A), suggesting that underlying serotype-pair risk differences are mediated through immune mechanisms not linked to quantitative measures of antibody titre. This may include proinflammatory cytokines induced by cross-reactive T cells or other factors^9–12,23^ that are parallel to the progression of humoral immunity.

We find that over and above the effect of the infecting serotype of the primary and secondary infections, disease risk is maximised at intermediate antigenic distances between the two infecting viruses (Figure 2B). We find that peak risk occurs when the antigenic distance between the primary and secondary infection is around 5.5 units (linear titre difference of 45.3). This provides evidence that the changing antigenic profile of circulating viruses within a serotype shifts the disease risk of the population. By translating antigenic distance to an approximation of the absolute titres generated from a primary infection when measured against different secondary infecting viruses, we estimate that peak risk occurs around an absolute titre of around 1:35 (Figure S3). These findings indicate a clear role of antibody titres in driving disease risk, with the specific pair of viruses that lead to primary and secondary infections altering the probability of disease. The magnitude of titres linked to peak risk is consistent with previous work^6,7^ that identified the association between intermediate titres averaged across four serotypes and disease risk in longitudinal cohort studies, where the specific antigenic properties of circulating viruses could not be considered due to limited prototype antigens used in the serologic assay.

**Figure 3.**
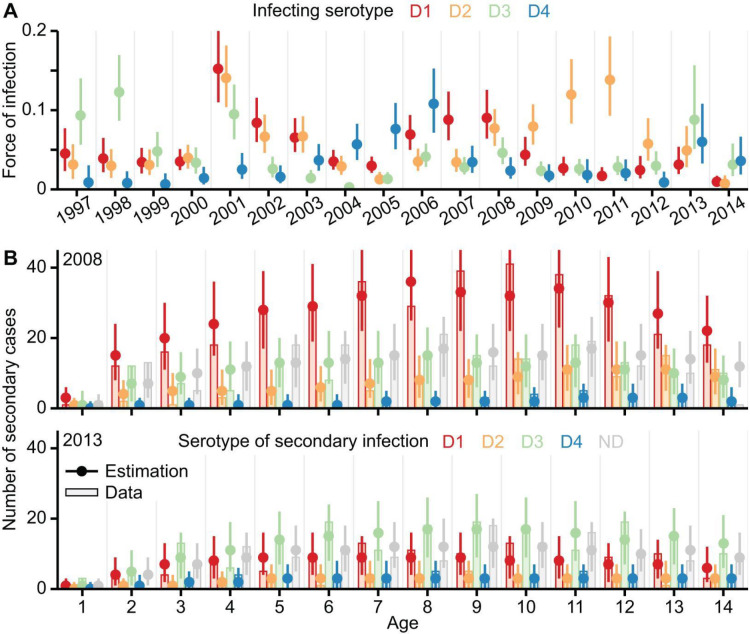
Estimated transmission intensity and model validation. **(A)** Annual force of infection by serotype, estimated using the full model. Dots and error bars indicate the median and 95% credible interval (CrI) of the posterior estimates, coloured by the infecting serotype. **(B)** Reconstruction of the yearly hospitalised secondary dengue cases by serotype and age. Results for 2008 and 2013, two years with distinct serotype patterns, are shown to illustrate the adequacy of model estimations. Figure S5 provides results for each year from 1997 to 2014. Vertical bars show the serotype and age-specific counts of secondary cases observed in our surveillance hospital. Dots and error bars indicate the median and 95% CrI of the corresponding case counts estimated by simulating the infection histories of individuals using posteriors inferred from the full model (Methods). The observed and estimated case counts are coloured by the identity of the secondary serotype. D1 to D4 indicate the secondary cases of each serotype. ND indicates cases without serotype information.

Using the full model, we find an average annual force of infection of 0.047 (95% CrI: 0.043–0.052) across the 18 years, with an overall steady decline over the time series (Figure S4), consistent with that found elsewhere^24^. Unlike previous efforts, our model also allows us to estimate serotype-specific changes in the force of infection (Figure 3A). We identify a pattern of cycling between the serotypes, with a mean period of 6.3 (95% CrI: 5.6–7.8) years between recurring epidemic peaks caused by the same serotype (Table S4). We find limited correlation between the annual force of infection of the different serotypes (mean absolute correlation of 0.17) (Table S5). We find a strong association between the annual force of infection and the mean number of hospitalised cases across all ages for each serotype in each year (mean correlation coefficient of 0.82; Table S6). To evaluate the adequacy of our model estimations, we reconstruct the serotype and age-specific counts of secondary cases hospitalised in each year from 1997 to 2014, using the posteriors of the full model to simulate the infection history of each person in Bangkok, Thailand (Methods). Results of our reconstruction are consistent with our original data of secondary cases, even for years with distinct serotype patterns (Figure 3B, Figure S5), and can explain 88.1% of the deviance in the annual serotype and age-specific case counts in our surveillance hospital.

## Evolution of population disease risk by year and age

**Figure 4.**
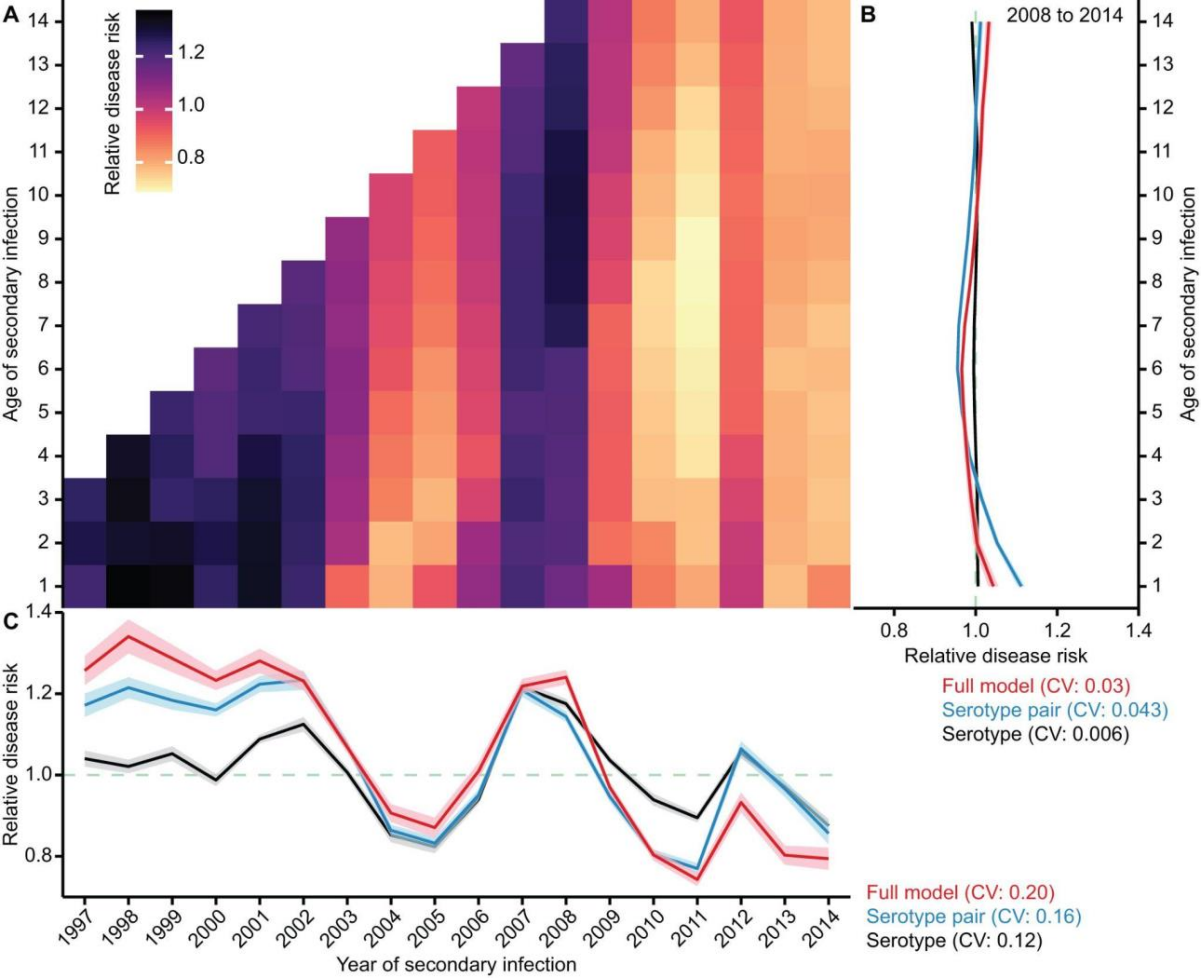
Evolution of population disease risk. **(A)** Relative disease risk by year and age with respect to the overall average of disease risks across all years and ages in the study. The estimation averages over 2,000 stochastic simulation realisations of the infection histories of individuals in Bangkok, using 40 randomly selected posteriors inferred from the full model. In each year from 1997 to 2007, older age groups without antigenic distance data were excluded from the analysis (Table S7). Colour in the heat map corresponds to the disease risk, which is estimated by adjusting the marginal probability of hospitalisation with the marginal probability of secondary infection for each cohort of individuals based on their year and age of secondary infections (Methods). **(B)** Mean relative disease risk for individuals of each age across years from 2008 to 2014. **(C)** Mean relative disease risk for individuals acquiring secondary infection in each year, which averages the estimated relative disease risk over different ages in the same year. In (B) and (C), colours indicate the estimates using different models, with the line and shaded regions indicating the respective mean and 95% confidence interval.

We use our full model to capture the changing disease risk for individual birth cohorts. We compare the risk of hospitalisation among those experiencing a secondary infection to focus on the role of antigenic properties of the circulating viruses in driving disease risk, rather than the role of extrinsic factors (e.g., climate factors) (Methods). We find that the relative risk of hospitalisation following a secondary infection can be up to 1.4 times as high for some age groups and years, as compared to the overall mean risk of hospitalisation from a secondary DENV infection across all age groups and years in the study (Figure 4A). It also shows a distinct pattern beyond the empirical analysis using secondary dengue cases observed in the hospital alone (Figure S1).

We find that overall, the risk of disease from a secondary infection is much less variable across age groups within a year (Figure 4B) than across years (Figure 4C). To assess how the antigenic properties of the circulating viruses and the serotype-specific epidemic patterns can drive the changing risk profile in the population by year, we compare the variability in disease risks by year across the different models. We find a greater year-to-year variability in underlying disease risk when using both the virus and serotype information to estimate disease risks (coefficient of variation of 0.20 for the full model; Figure 4C), compared to using only serotype information to estimate disease risks (coefficient of variation of 0.12 and 0.16 for the other two serotype-based models; Figure 4C). Therefore, alongside the primary and secondary infecting serotypes, inter-annual variation in the antigenic properties of the circulating viruses has important contributions to the changes in disease risk (Figures S8–9).

To assess the extent to which the fluctuating risk of disease by year is driven by the cyclical epidemic nature of the four dengue serotypes as shown in Figure 3A, we repeated the above analysis but instead assumed that all serotypes circulate with the same constant force of infection across all years, with the antigenic properties of the circulating viruses remaining the same. In this scenario, we find that the disease risk from a secondary infection has substantially weakened variance across age groups and years (Figure S6). These comparisons suggest that the cyclical epidemic patterns of the four serotypes mediate both the cyclical antigenic priming of individuals and their subsequent secondary infections, which in turn propel the development of severe dengue. The extent of such disease progression depends on both the specific serotype and the antigenic characteristics of the sequential infecting viruses.

We further use the full model to assess the respective importance of antigenic imprinting or secondary infection in determining disease risk. In a hypothetical scenario, if disease risk from a secondary infection depends solely on the nature of the primary infecting virus, individuals with the same primary infecting virus would have no variation in the risk of disease across subsequent years, regardless of the secondary infecting virus. Conversely, if disease risk depends solely on the secondary infecting virus, individuals with the same initial infecting virus would have high variability in the year-to-year risk of disease, as the primary infecting virus becomes irrelevant. We find that the variation in the risk of subsequent disease is comparable between individuals with a specific primary infecting virus across different possible secondary viruses (coefficient of variation of 0.86 (95% CrI: 0.27 to 1.68)) and those with a specific secondary infecting virus across different possible primary infecting viruses (coefficient of variation of 0.69 (95% CrI: 0.24 to 1.38)). This shows an approximately equal contribution of the primary and secondary infecting virus to the risk of disease.

## Discussion

Our findings challenge the prevailing paradigm that the introduction of a new serotype is responsible for shifts in disease risk in a population. Instead, our findings suggest a more nuanced picture where the specific impact of a new virus on patterns of disease will depend on both the characteristics of that virus and the population immunity derived from previous circulations of antigenically different viruses, with the impact of a particular virus being potentially different across populations with different exposure histories. Our findings are consistent with the concept of original antigenic sin and other related hypotheses, such as immune imprinting, where an individual’s first infection largely determines which viruses they are most protected against and contributes to future disease risk by other related viruses^15,25,26^. Our approach to bring together sequence data, antigenic maps, and surveillance data into integrative frameworks will be relevant to other antigenically variable pathogens, including influenza, SARS-CoV-2, and norovirus^1,2,27^. They also provide a route to determining the evolutionary pathways that viruses take in adapting to local immunity.

Our findings support a role for the standardised antigenic characterisation of circulating DENV strains against reference antisera mounting immunity to a diverse set of DENV, as is done with influenza^28^. By characterising the virus-specific immune history of populations, we could quantify setting-specific risk profiles to existing and emerging strains. Systematically quantifying the properties of human sera against previous and current circulating strains will help discern the changes in immune profiles and pathogenesis after natural infection and vaccination^29–31^. This will become increasingly important as dengue vaccines begin to get used^12,17,18^. The antigenic distance between vaccine strains and locally circulating strains may help explain underlying differences in the efficacy of vaccines across populations, with the potential of a long-term goal of tailored vaccines based on local antigenic profiles.

## Methods

### Hospitalised dengue case data.

Linelist data of hospitalised dengue cases was from the Queen Sirikit National Institute of Child Health (QSNICH) in Bangkok, Thailand. QSNICH is the only public children’s hospital at the tertiary level in Bangkok that serves dengue cases in children requiring hospitalisation^19^. All suspected dengue cases that underwent hospitalisation at QSNICH were tested using reverse transcriptase polymerase chain reaction or IgM/IgG serology at the Armed Forces Research Institute of Medical Sciences^19^. Primary or secondary infection was determined using dengue hemagglutination inhibition assay and/or dengue IgM/IgG capture enzyme-linked immunosorbent assay (ELISA)^19^. The infecting serotype was determined using serotype-specific PCR and/or antigen-capture ELISA^19^. From 1997 to 2014, there were 11,918 secondary cases, 2,464 primary cases, and 899 cases without infection parity. We only used secondary cases. The infant cases (N=38) were excluded to avoid the influence of maternally-derived antibodies^9,32^. Only 335 (2.8%) secondary cases were with age above 14, partly because young adults tended to attend general hospitals. Using the age groups between 1 and 14, we identified 11,546 secondary cases in QSNICH from 1997 to 2014. The yearly age-specific population data in Bangkok was retrieved from^33^.

### Full genome sequencing of dengue viruses.

The selection criteria of QSNICH serum samples for virus isolation and full genome sequencing were described previously^13,20^. In total 1,848 dengue viruses isolated from QSNICH patients over the 21-year period from 1994 to 2014, including 622 DENV-1, 438 DENV-2, 424 DENV-3, and 364 DENV-4 strains, were sequenced at Walter Reed Army Institute of Research (WRAIR), using Illumina MiSeq or Roche 454 sequencing. An additional 739 dengue viruses isolated from other locations in Thailand were also underwent full genome sequencing at WRAIR. Assembly of consensus genomes and construction of consensus sequences were previously described^13,20^.

The Maximum Clade Credibility phylogenies of all strains from the same serotype, based on full genome sequences, were built using BEAST v1.10.4^34^, under a HKY codon substitution model^35^, a relaxed lognormal clock model^36^, and a skygrid population size model^37^. Three independent chains were run for each serotype, with parameters sampled every 10,000 iterations. Runs were optimised using the GPU BEAGLE 4 library^38^. For each serotype, combined chains were manually checked for convergence using the Tracer software^39^, with effective sampling size (ESS) values > 200. For DENV-2 to DENV-4, the chains were run for 1 to 1.5 billion iterations, where we manually removed the initial 10% iterations as burn-in depending on the serotype. For DENV-1, considering the large size of the sequence dataset, we allowed some ESS to be between 150 and 200 to avoid prohibitive computational time. Two chains were run for 900 millions iterations with the initial 18% iterations as burn-in, while the third chain was run for 1.2 billion iterations with the initial 40% iterations as burn-in to correct for convergence issues.

### Antigenic characterisation of the representative dengue viruses isolated from Bangkok.

Among the 1,848 dengue viruses isolated from Bangkok, 348 antigenically representative strains, including 87 DENV-1, 80 DENV-2, 90 DENV-3, and 91 DENV-4 strains, were selected for the antigenic measurements using PRNT assay. The titration experiments used a panel of 20 antisera from African green monkeys^13^. The selection criteria of the representative viruses and antisera for antigenic measurements and the adjustment of titres for experimental conditions were described previously^13^. We constructed the original antigenic map of dengue viruses using the approach of antigenic cartography^40,41^, as described previously^13^.

DENV-1 in 2003, 2008 and 2009, DENV-2 in 2009, and DENV-3 in 1999 and 2007 only had sequencing data but no antigenic measurements. DENV-2 in 2008 did not have both sequences and antigenic measurements. To provide map coordinates for each of these viruses uncharted in the original antigenic map, we developed the following data curation algorithm. First, consider DENV strains of serotype $s_{i}$ isolated at year T, with sequencing data but no antigenic data. We identified all antigenically characterised viruses of the same serotype $s_{i}$ isolated between year T-2 and T+2. For each sequenced virus of serotype $s_{i}$ isolated at year T, we identified the genetically closest virus from this subset of antigenically characterised viruses and gave the sequenced virus the same coordinates. If multiple sequenced viruses of serotype $s_{i}$ isolated at year T were given the same map coordinates, they were merged as a single virus. Then, we identified all antigenically characterised viruses of DENV-2 isolated between 2006 and 2010. For each pair of identified viruses, we used their map coordinates to calculate the corresponding antigenic distance. We excluded those virus pairs with antigenic distance greater than 0.75 (i.e., mean antigenic distance due to the variability of PRNT measurements^42^). We partitioned the remaining viruses into four groups using the k-means clustering^40^, and then aggregated viruses of each group into a single virus by averaging their map coordinates. These procedures introduced 48 additional viruses into the original antigenic map.

Our study used the 3D antigenic map to conduct all the inference and analyses, and 2D antigenic map only for the visualisation of Figure 1B. To ensure the coverage of antigenic distance data for all years and ages of secondary dengue cases hospitalised in QSNICH, we excluded those older age groups with no antigenic distance data (Table S7). The final case data aligned with the antigenic distance data contains 6,903 hospitalised secondary cases, with 69.7% of cases diagnosed with the infecting serotype of the secondary infection.

### Modelling dengue virus infection and disease process.

Consider an individual of age $a_{T}$ at year T. Let k=1 and k=2 denote the primary and secondary infection, respectively. Let $P_{Infection}\left(\underset{k=1}{s_{j},Y},\;\underset{k=2}{\underset{︸}{s_{i},T,a_{T}}}\right)$ be the probability that this individual acquires primary infection with serotype $s_{j}$ at year Y and secondary infection with serotype $s_{i}$ at year T. Given the trajectory of these two sequential infections, this individual becomes a diseased case in our surveillance hospital with probability $P_{Disease}^{k=2}\left(\underset{k=1}{s_{j},Y},\;\underset{k=2}{s_{i},T}\right)$.

We only analyse the secondary dengue cases observed in hospital, with no information about their primary infections. By considering the serotype, virus, and age of each individual’s primary infection as latent variables, the probability that an individual of age $a_{T}$ at year T is hospitalised as a severe dengue case due to the secondary infection with serotype $s_{i}$ in that year is given by:

(1) $$H_{Disease}^{k=2}\left(s_{i},T,a_{T}\right)=\sum_{Y=T-a_{T}}^{T-1}\sum_{j\neq i}P_{Infection}\left(\underset{k=1}{s_{j},Y},\;\underset{k=2}{\underset{︸}{s_{i},T,a_{T}}}\right)\cdot P_{Disease}^{k=2}\left(\underset{k=1}{s_{j},Y},\;\underset{k=2}{s_{i},T}\right).$$

### Infection process.

Given the above-mentioned trajectory of primary and secondary infections, the individual’s birth year is T-Y, and the age at the year of primary infection is $a_{Y}=a_{T}-T+Y$. The probability for the occurrence of this individual infection trajectory is given by:

(2) $$P_{Infection}\left(\underset{k=1}{s_{j},Y},\;\underset{k=2}{\underset{︸}{s_{i},T,a_{T}}}\right)=P_{Infection}^{k=1}\left(s_{j},Y,a_{Y}\right)\cdot P_{Infection}^{k=2}\left(s_{i},T,a_{T}|s_{j},Y,a_{Y}\right)\text{,}$$

where the two terms on the right-hand side are explained as follows.

#### Primary infection.

The first term $P_{Infection}^{k=1}\left(s_{j},Y,a_{Y}\right)=\left(\prod_{Z=T-a_{T}}^{Y-1}P_{Escape}^{k=0}\left(Z\right)\right)\cdot P_{k\to 1}\left(s_{j},Y\right)$ is the joint probability that an individual of age $a_{T}$ at year T remains fully susceptible before year Y and then gets primary infection with serotype $s_{j}$ at year Y. Specifically, $P_{Escape}^{k=0}(Z)=\exp\left(-\sum_{i}\lambda \left(s_{i},Z\right)\right)$ is the probability that a naïve individual escapes all four serotypes at year Z, and $P_{k\to 1}\left(s_{j},Y\right)=\left(1-\exp\left[-\lambda \left(s_{j},Y\right)\right]\right)\cdot \exp\left(-\sum_{m\neq j}\lambda \left(s_{m},Y\right)\right)$ is the probability that a naïve individual gets primary infection with serotype $s_{j}$ at year Y.

The force of infection^43^ (FOI), $\lambda \left(s_{j},Y\right)$, is the rate at which a naïve individual gets primary infection with serotype $s_{j}$ at year Y. The FOI captures the influence of extrinsic factors (e.g., local climate conditions, mosquito abundance, and host-mosquito interactions) on dengue epidemics in human population.

#### Secondary infection.

The second term in Eq. (2)

$$P_{Infection}^{k=2}\left(s_{i},T,a_{T}|s_{j},Y,a_{Y}\right)=\left(\prod_{X=Y+1}^{T-1}\;\;P_{Escape}^{k=1}\left(X|s_{j},Y\right)\right)\cdot P_{k\to 2}\left(s_{i},T|s_{j},Y\right)$$

is the probability that an individual of age $a_{T}$ at year T gets secondary infection with serotype $s_{i}$ at year T, conditional on the primary infection with heterotypic serotype $s_{j}$ at year Y. Specifically, $P_{Escape}^{k=1}\left(X|s_{j},Y\right)=\exp\left(-\sum_{m\neq j}\Lambda_{k=2}\left(s_{m},X|s_{j},Y\right)\right)$ is the conditional probability that after the primary infection with serotype $s_{j}$ at year Y, the individual escapes secondary infection with any of the other three serotypes, $s_{m}\neq s_{j}$, at year X∈[Y+1,T-1]. And $P_{k\to 2}\left(s_{i},T|s_{j},Y\right)=\left(1-\exp\left[-\Lambda_{k=2}\left(s_{i},T|s_{j},Y\right)\right]\right)\cdot \exp\left(-\sum_{h\neq j,i}\Lambda_{k=2}\left(s_{h},T|s_{j},Y\right)\right)$ is the conditional probability of getting secondary infection with serotype $s_{i}$ at year T, given the previous primary infection with serotype $s_{j}$ at year Y.

#### Temporary cross-protection.

The immune responses elicited by primary infection generate both serotype-specific antibodies and cross-reactive antibodies^11,12^. The serotype-specific antibodies often maintain at a relatively high concentration over time, providing life-long protection against homotypic reinfections^10,11^. Following primary infection, cross-reactive antibodies can provide temporary cross-protection to reduce the risk of getting heterotypic infections. However, the waning of immunity over time may reduce the cross-reactive antibodies to a low concentration and give rise to sub-neutralising antibody titres that enhance the risk of severe disease^6,7^.

Therefore, we make two assumptions: (1) The homotypic reinfections are unlikely to occur or be observed in hospitals, and hence excluded from our analysis; and (2) the temporary cross-protection reduces the susceptibility to heterotypic infections by a factor of 1-ξ in the first year following primary infection. To account for the effect of temporary cross-protection, we introduce the effective FOI to adjust the risk of infection at year X≥Y+1 for individuals with primary infection at year Y, as below:

$$\Lambda_{k=2}\left(s_{m},X|s_{j},Y\right)=\left\{\begin{array}{r} \xi \cdot \lambda \left(s_{m},X\right)\cdot I\left\{s_{m}\neq s_{j}\right\},\;\text{if}\;X=Y+1\\ \lambda \left(s_{m},X\right)\cdot I\left\{s_{m}\neq s_{j}\right\},\;\text{if}\;X>Y+1 \end{array}\right.,$$

where I{⋅} is the indicator function, and ξ is the relative change in FOI due to temporary cross-protection.

### Full model of dengue disease process.

Given the infection trajectory of primary infection with serotype $s_{j}$ at year Y and secondary infection with serotype $s_{i}$ at year T, the probability of becoming a dengue case in hospital is given by:

(3) $$P_{Disease}^{k=2}\left(s_{j},Y,s_{i},T\right)=\rho_{Disease}^{k=2}\left(s_{j},s_{i}\right)\cdot E\left[\psi_{Disease}^{k=2}\left(D\left(v_{j},v_{i}\right)|s_{j},Y,s_{i},T\right)\right],$$

which incorporates the following two components.

#### Probability of disease by serotype-pair.

Existing evidence from immunopathogenesis and serology studies suggests that the risk of severe dengue following a secondary infection may differ depending on the infecting serotypes of the primary and secondary infections^9–11,23^. To account for this effect in Eq. (3), we use the term $\rho_{Disease}^{k=2}\left(s_{j},s_{i}\right)$ to characterise the probability of disease given the sequential infections with primary serotype $s_{i}$ and then secondary serotype $s_{i}$.

#### Relative probability of disease by antigenic distance.

Alongside the effect of the two specific serotypes causing primary and secondary infections, we further consider the influence of the specific pair of viruses that are potentially responsible for an individual’s primary and secondary infections on the probability of disease from a secondary infection. This is captured by the term $E\left[\psi_{Disease}^{k=2}\left(D\left(v_{j},v_{i}\right)|s_{j},Y,s_{i},T\right)\right]$ in Eq. (3).

Let $n_{v}\left(s_{j},Y\right)$ be the number of viruses within serotype $s_{j}$ at year Y. If the primary infection is caused by virus $v_{j}\in \left[1,n_{v}\left(s_{j},Y\right)\right]$ from serotype $s_{j}$ at year Y and secondary infection is caused by virus $v_{i}\in \left[1,n_{v}\left(s_{i},T\right)\right]$ from serotype $s_{i}$ at year T, then the relative probability of disease in terms of the antigenic distance $D\left(v_{j},v_{i}\right)$ separating these two viruses is described by:

(4) $$\psi_{Disease}^{k=2}\left(D\left(v_{j},v_{i}\right)|s_{j},Y,s_{i},T\right)=\sum_{\ell =1}^{L+d-1}\alpha_{\ell }\cdot B_{\ell }^{d}\left(D\left(v_{j},v_{i}\right)\right)\text{,}$$

which uses the B-spline method^44^ to convert the antigenic distance into the corresponding relative probability of disease. Here, $B_{\ell }^{d}\left(D\left(v_{j},v_{i}\right)\right)$ represents the B-spline basis functions of the antigenic distance, and $\alpha_{\ell }$ the corresponding B-spline coefficients (see below for more details).

To incorporate uncertainty in the virus causing each infection, we average the virus-specific relative probability of disease, as described by Eq. (4), over all possible pairs of viruses that could result in the given trajectory of primary and secondary infections as follows:

$$E\left[\psi_{Disease}^{k=2}\left(D\left(v_{j},v_{i}\right)|s_{j},Y,s_{i},T\right)\right]=\frac{1}{n_{v}\left(s_{j},Y\right)n_{v}\left(s_{i},T\right)}\sum_{v_{j}=1}^{n_{v}\left(s_{j},Y\right)}\;\sum_{v_{i}=1}^{n_{v}\left(s_{i},T\right)}\;\psi_{Disease}^{k=2}\left(D\left(v_{j},v_{i}\right)|s_{j},Y,s_{i},T\right)$$

#### B-spline method.

Let d be the degree of a polynomial. Let $\tau_{1},\tau_{2},\ldots ,\tau_{L}$ be a non-decreasing sequence of knots, with two boundary knots bracketing the range of data. A spline function can be constructed as:

$$f(X)=\sum_{\ell =1}^{L+d-1}\;a_{\ell }\cdot B_{\ell }^{d}(X),a_{\ell }\in ,$$

where $B_{\ell }^{d}$ are a set of basis functions, and $a_{\ell }$ are the spline coefficients^44^. Since the two boundary knots bracket the range of data, the family of basis functions has L+d-1 members.

B-spline basis is a commonly used spline basis. Any spline function of a given degree *d* can be expressed as a linear combination of B-splines of that degree. To define B-splines of degree d covering the whole span of the knots, the original sequence of knots is extended as: $\underset{d\;\text{times}}{\underbrace{\tau_{1},\ldots ,\tau_{1}}},\underset{\text{original}\;\text{knots}}{\underbrace{\tau_{1},\tau_{2},\ldots ,\tau_{L}}},\underset{d\;\text{times}}{\underbrace{\tau_{L},\ldots ,\tau_{L}}}$. Then, let $B_{\ell }^{d}$ be the ℓ-th member of the family of B-splines of degree d, with 1≤ℓ≤L+d-1. B-splines of degree d=0 are a set of piecewise constant functions, defined as:

$$B_{\ell }^{d=0}(x)=\left\{\begin{array}{rr} 1, & \;\text{if}\;\tau_{\ell }\leq x<\tau_{\ell +1}\\ 0, & \;\text{otherwise}\; \end{array}.\right.$$

B-splines of higher degree d>0 are defined recursively:

$$B_{\ell }^{d}(x)=w_{\ell }^{d}(x)\cdot B_{\ell }^{d-1}(x)+\left(1-w_{\ell +1}^{d}(x)\right)\cdot B_{\ell +1}^{d-1}(x),$$

where

$$w_{\ell }^{d}(x)=\left\{\begin{array}{c} \frac{x-\tau_{\ell }}{\tau_{\ell +d}-\tau_{\ell }},\;\text{if}\;\tau_{\ell }\neq \tau_{\ell +d}\\ 0,\;\;\text{otherwise}\; \end{array}.\right.$$

In our case, let $\Theta \left\{D\left(v_{j},v_{i}\right)\right\}$ be the whole dataset of the antigenic distance between the two viruses that are responsible for an individual’s primary and secondary infections. We partition the range of antigenic distance data $\Theta \left\{D\left(v_{j},v_{i}\right)\right\}$ using a non-decreasing sequence of knots, $\tau_{1},\tau_{2},\ldots ,\tau_{L}$. The boundary knots $\tau_{1}$ and $\tau_{L}$ indicate the minimum and maximum antigenic distance, and the interior knots $\tau_{2},\ldots ,\tau_{L-1}$ partition the range of antigenic distance data into L-1 bins with equal quantiles. Our disease model described by Eq. (4) is built with cubic polynomials with degree d=3. Model comparison results suggest the use of L=10 knots in the full model to achieve sound fitting performance (Table S2).

### Serotype-pair model of dengue disease process.

We consider an alternative model where the probability of disease from a secondary infection depends on the serotype of both the primary and secondary infection:

(5) $$P_{Disease}^{k=2}\left(s_{j},Y,s_{i},T\right)=\rho_{Disease}^{k=2}\left(s_{j},s_{i}\right).$$

### Serotype model of dengue disease process.

We further consider a simplified model where the probability of disease from a secondary infection only depends on the identity of the secondary serotype:

(6) $$P_{Disease}^{k=2}\left(s_{j},Y,s_{i},T\right)=\rho_{Disease}^{k=2}\left(s_{i}\right).$$

### Accounting for a subset of cases with no serotype information.

In our hospital linelist case data, serotype information is not available for 30.3% of secondary cases across all years. Let η(T) be the proportion of secondary cases having serotype information in hospital at year T. We calculate η(T) as the ratio between the number of secondary cases having serotype information in year T and the total number of secondary cases observed in year T.

The probability that an individual of age $a_{T}$ at year T acquiring secondary infection with serotype $s_{i}$ at year T becomes a severe case with known serotype in hospital is given by:

$$H_{i}^{k=2}\left(s_{i},T,a_{T}\right)=\eta \left(T\right)\cdot H_{Disease}^{k=2}\left(s_{i},T,a_{T}\right).$$

The probability that an individual of age $a_{T}$ at year T acquiring secondary infection with serotype $s_{i}$ at year T becomes a severe case in hospital but with no serotype information is given by:

$$H_{NA}^{k=2}\left(s_{i},T,a_{T}\right)=\left[1-\eta \left(T\right)\right]\cdot H_{Disease}^{k=2}\left(s_{i},T,a_{T}\right).$$

Let $N\left(T,a_{T}\right)$ be the population size for individuals of age $a_{T}$ at year T in Bangkok. The expected number of secondary cases with known serotype $s_{i}$ in our hospital is given by:

(7) $$E\left[I_{k=2}\left(s_{i},T,a_{T}\right)\right]=N\left(T,a_{T}\right)\cdot H_{i}^{k=2}\left(s_{i},T,a_{T}\right).$$

The expected number of secondary cases with no serotype information in our surveillance hospital is given by:

(8) $$E\left[I_{k=2}\left(s_{NA},T,a_{T}\right)\right]=N\left(T,a_{T}\right)\cdot \sum_{i}H_{NA}^{k=2}\left(s_{i},T,a_{T}\right).$$

### Likelihood function.

Let $I_{k=2}\left(s_{i},T,a_{T}\right)$ be the number of secondary cases within individuals of age $a_{T}$ at year T, who were observed with serotype $s_{i}$ in our surveillance hospital. Let $I_{k=2}\left(s_{NA},T,a_{T}\right)$ be the number of secondary cases within individuals of age $a_{T}$ at year T, who did not have serotype information in our surveillance hospital.

To account for the potential overdispersion in the hospital case data^33^, the likelihood of the yearly age-specific secondary cases observed with serotype $s_{i}$ follows a negative binomial distribution, given by:

$$L\left(s_{i},T,a_{T}\right)∼\mathrm{Neg}\mathrm{Binomial}\left(I_{k=2}\left(s_{i},T,a_{T}\right)|E\left[I_{k=2}\left(s_{i},T,a_{T}\right)\right],\;\phi \left(s_{i},T,a_{T}\right)\right),$$

where $\phi \left(s_{i},T,a_{T}\right)=\frac{p_{NB}}{1\;-\;p_{NB}}\cdot E\left[I_{k=2}\left(s_{i},T,a_{T}\right)\right]$ is the dispersion parameter, with $p_{NB}$ being the probability of success per trial in the negative binomial distribution.

Similarly, the likelihood of the yearly age-specific secondary cases with no serotype information is given by:

$$L\left(s_{NA},T,a_{T}\right)∼\mathrm{Neg}\mathrm{Binomial}\left(I_{k=2}\left(s_{NA},T,a_{T}\right)|E\left[I_{k=2}\left(s_{NA},T,a_{T}\right)\right],\;\phi \left(s_{NA},T,a_{T}\right)\right),$$

where $\phi \left(s_{NA},T,a_{T}\right)=\frac{p_{NB}}{1\;-\;p_{NB}}\cdot E\left[I_{k=2}\left(s_{NA},T,a_{T}\right)\right]$.

The overall log likelihood of the entire hospital data of secondary dengue cases is given by:

$$\sum_{T=1997}^{2014}\sum_{a_{T}=1}^{a_{T}^{U}}\sum_{i=1}^{4}\log\left(L\left(s_{i},T,a_{T}\right)\right)+\sum_{T=1997}^{2014}\sum_{a_{T}=1}^{a_{T}^{U}}\log\left(L\left(s_{NA},T,a_{T}\right)\right),$$

where $a_{T}^{U}$ is the oldest age analysed for year T, for which individuals older than $a_{T}^{U}$ in year T are excluded as they do not have antigenic distance data (Table S7).

### Model fitting: Prior settings and diagnostics.

We infer all model parameters in a Bayesian framework using Markov Chain Monte Carlo (MCMC) method with Hamiltonian Monte Carlo sampling^45^. We implement all inference models using CmdStanR^46^ and RStan^47^ packages in R. We fit the model using the following weakly informative priors^48^:

1. Serotype-specific FOI per year was sampled with the following prior distribution: $\lambda \left(s_{i},T\right)∼0.08+0.08*normal(0,1)$, with $0\leq \lambda \left(s_{i},T\right)\leq 0.4$.
2. Relative change in FOI due to temporary cross-protection was sampled with the prior: ξ∼0.5+0.5*normal⁡(0,10), with 0≤ξ≤1.
3. Based on our previous estimates of the case reporting rates in Thailand^33^, the probability of disease by the serotype of the primary and secondary infection was sampled using below two steps:
  First, DENV-1 followed by DENV-3 infection was regarded as the reference pair, with the corresponding probability of disease sampled with the prior:
$$\rho_{Disease}^{k=2}\left(s_{j=1},s_{i=3}\right)∼0.01+0.005*normal(0,1)\;\text{with}\;0\leq \rho_{Disease}^{k=2}\left(s_{j=1},s_{i=3}\right)\leq 0.05.$$
  Then, the probability of disease for each of the other 11 serotype pairs relative to that of the reference pair was sampled with the prior distribution:
$$\frac{\rho_{Disease}^{k=2}\left(s_{j},s_{i}\right)}{\rho_{Disease}^{k=2}\left(s_{j=1},s_{i=3}\right)}∼1+normal(0,0.5)\;\text{with}\;0\leq \frac{\rho_{Disease}^{k=2}\left(s_{j},s_{i}\right)}{\rho_{Disease}^{k=2}\left(s_{j=1},s_{i=3}\right)}\leq 10.$$
4. B-spline coefficients for the relative probability of disease by antigenic distance were sampled with the prior: $\alpha_{\ell }∼1+2*normal(0,1)$, with $\alpha_{\ell }\geq 0$.
5. Success probability per trial in the negative binomial distribution was sampled with the prior: $p_{NB}∼0.5+0.5*normal(0,10)$, with $0\leq p_{NB}\leq 1$.

To fit each model, we use four independent chains with random initialisations. Each chain was run for 45,000 iterations with the initial 30,000 iterations as warm-up. We validate the convergence of MCMC chains using the trace plot and Gelman-Rubin R statistic^45^. We obtain the posterior samples using a thinning interval of 50.

### Reconstruction of the serotype and age-specific counts of hospitalised secondary cases in each year from 1997 to 2014.

Using an individual-based stochastic simulation model parameterised with the posteriors inferred from the full model, we simulate the infection and disease process of each person in Bangkok over the study period. We randomly select 40 posterior samples inferred from the full model, each of which is simulated with 100 stochastic realisations. This simulator has below components.

#### Pre-calculation of the probability of disease.

Before simulations, we first use Eq. (3) to calculate the probability of disease for each possible infection trajectory, $P_{Disease}^{k=2}\left(s_{j},Y,s_{i},T\right)$, based on the specific serotype and year of the primary and secondary infections.

#### Initial demographics.

The first year in simulation is set to 1994. Based on the age-specific population data in Bangkok^33^, a total of 76,688 newborns fully susceptible to all four serotypes are introduced at the start of each simulation realisation.

#### New primary and secondary infections generated in each simulation year.

At the start of each year T, we identify all seronaive individuals fully susceptible to all four serotypes and all exposed individuals only having primary infections before year T.

Then, we simulate the occurrence of primary infections at year T. The probability that each seronaive individual gets primary infection with serotype $s_{j}$ at year T is given by: $P_{k\to 1}\left(s_{j},T\right)=\left(1-\exp\left[-\lambda \left(s_{j},T\right)\right]\right)\cdot \exp\left(-\sum_{m\neq j}\lambda \left(s_{m},T\right)\right)$. The probability that each seronaive individual escapes all four serotypes to remain fully susceptible in year T is given by $1-\sum_{j=1}^{4}P_{k\to 1}\left(s_{j},T\right)$. Therefore, for each seronaive individual, the process of getting primary infection with one serotype or escaping all four serotypes at year T is simulated using a multinomial distribution with probabilities: $\left\{P_{k\to 1}\left(s_{j},T\right),\forall s_{j};1-\sum_{j=1}^{4}P_{k\to 1}\left(s_{j},T\right)\right\}$.

Then, we simulate the occurrence of secondary infections at year T. The probability that each exposed individual only having a primary infection with serotype $s_{j}$ at year Y acquires secondary infection with a heterotypic serotype $s_{i}$ at year T is given by:$P_{k\to 2}\left(s_{i},T|s_{j},Y\right)=\left(1-\exp\left[-\Lambda_{k=2}\left(s_{i},T|s_{j},Y\right)\right]\right)\cdot \exp\left(-\sum_{h\neq j,i}\Lambda_{k=2}\left(s_{h},T|s_{j},Y\right)\right)$. The probability that each exposed individual further escapes the secondary infection at year T is given by: $1-\sum_{i\neq j}P_{k\to 2}\left(s_{i},T|s_{j},Y\right)$. Therefore, for each individual exposed only once before year T due to primary infection with serotype $s_{j}$ at year Y, the process of getting secondary infection with a heterotypic serotype $s_{i}\neq s_{j}$ or escaping all three heterotypic serotypes at year T is simulated using a multinomial distribution with probabilities: $\left\{P_{k\to 2}\left(s_{i},T|s_{j},Y\right),\forall s_{i}\neq s_{j};\;1-\sum_{i\neq j}P_{k\to 2}\left(s_{i},T|s_{j},Y\right)\right\}$.

#### New hospitalisation of secondary cases per year.

The probability that an individual having a primary infection with serotype $s_{j}$ at year Y and a secondary infection with serotype $s_{i}$ at year T becomes severe case in hospital with known serotype is given by: $\eta (T)\cdot P_{Disease}^{k=2}\left(s_{j},Y,s_{i},T\right)$. The probability that an individual having the same infection trajectory becomes a severe case in hospital but with no serotype information is given by: $[1-\eta (T)]\cdot P_{Disease}^{k=2}\left(s_{j},Y,s_{i},T\right)$. Therefore, the disease process of each secondary infection at year T is simulated using a multinomial distribution with probabilities:

$$\left\{\eta \left(T\right)\cdot P_{Disease}^{k=2}\left(s_{j},Y,s_{i},T\right);\;[1-\eta (T)]\cdot P_{Disease}^{k=2}\left(s_{j},Y,s_{i},T\right);1-P_{Disease}^{k=2}\left(s_{j},Y,s_{i},T\right)\right\}.$$

#### Update of demographics.

The following procedures run in succession at the end of each year T: (1) The age of each individual increases by one year; (2) individuals older than $a_{T}^{U}$ (i.e., oldest age analysed for year T, Table S7) are removed; (3) each remaining individual is randomly selected to remove according to the death rate at year T; and (4) newborns are generated according to the age-specific population data for the subsequent year T+1.

### Characterisation of the evolution of population disease risk by year and age.

We extend the above simulation approach to estimate the changing disease risk for individual birth cohorts over year and age. We compare the estimated disease risks using the full model, the serotype-pair model, and the serotype model. With the parameters inferred from each model, we randomly select 40 posterior samples and use each of them to simulate 100 stochastic realisations. Each realisation first simulates the infection process of the primary and secondary infections for each person in Bangkok across the study period, and then calculates the disease risk by adjusting the marginal probability of hospitalisation with the marginal probability of secondary infection for each cohort of individuals based on their year and age of secondary infections. This simulator has the following components:

#### Initial demographics.

The first year in simulation is set to 1994. Based on the age-specific population data^33^, a total of 76,688 newborns fully susceptible to all four serotypes are introduced at the start of each simulation realisation.

#### Infection process of each individual.

At the start of each year T, we identify all seronaive individuals fully susceptible to all four serotypes and all exposed individuals only having primary infections before year T.

Then, we simulate the occurrence of primary infections at year T. For each seronaive individual, the process of getting primary infection with one serotype or escaping all four serotypes at year T is simulated using a multinomial distribution with probabilities:

$$\left\{P_{k\to 1}\left(s_{j},T\right),\forall s_{j};1-\sum_{j=1}^{4}P_{k\to 1}\left(s_{j},T\right)\right\}.$$

All viruses from the same serotype co-circulating at the same year are assumed to be equally transmissible. For each primary infection with a given serotype $s_{j}$ at year T, the infecting virus is randomly chosen from all co-circulating viruses of the same serotype with probability:

$$\frac{1}{n_{v}\left(s_{j},T\right)}.$$

Then, we simulate the occurrence of secondary infections at year T. For each individual exposed only once before year T due to primary infection with serotype $s_{j}$ at year Y, the process of getting secondary infection with a heterotypic serotype $s_{i}\neq s_{j}$ or escaping all three heterotypic serotypes at year T is simulated using a multinomial distribution with probabilities:

$$\left\{P_{k\to 2}\left(s_{i},T|s_{j},Y\right),\forall s_{i}\neq s_{j};1-\sum_{i\neq j}P_{k\to 2}\left(s_{i},T|s_{j},Y\right)\right\}.$$

For each secondary infection with a given serotype $s_{i}$ at year T, the infecting virus is randomly chosen from all co-circulating viruses of the same serotype with probability: $\frac{1}{n_{v}\left(s_{i},T\right)}$.

At the end of each simulation year, all individuals have a one-year increase in age, and then newborns are generated according to the age-specific population data in Bangkok. The removal of individuals due to death or ageing is not considered, due to the need of recording the simulated individual infection trajectories for the subsequent analysis. Our simulation records the following information of each individual with two infections: (1) The year of birth, (2) the infecting year, serotype, and virus of the primary and secondary infections, (3) the antigenic distance between the two viruses causing the primary and secondary infections, and (4) the relative probability of disease $\psi_{Disease}^{k=2}$ in terms of the antigenic distance between the two infecting viruses, calculated using Eq. (4).

#### Estimation of population disease risk by year and age.

After the simulation of all 21 years, we calculate the population disease risk based on the year and age of secondary infections. First, we calculate the marginal probability of secondary infection for individuals of age $a_{T}$ at year T by:

$$P_{Infection}^{k=2}\left(T,a_{T}\right)=\sum_{i=1}^{4}\sum_{Y=T-a_{T}}^{T-1}\sum_{j\neq i}P_{Infection}\left(\underset{k=1}{s_{j},Y},\;\underset{k=2}{\underset{︸}{s_{i},T,a_{T}}}\right)\text{,}$$

using the posterior estimation of the serotype-specific FOI per year and the effect of temporary cross-protection.

Then we calculate the marginal probability of hospitalisation for individuals of age $a_{T}$ at year T by

$$P_{Disease}^{k=2}\left(T,a_{T}\right)=\sum_{i=1}^{4}\sum_{Y=T-a_{T}}^{T-1}\sum_{j\neq i}P_{Infection}\left(\underset{k=1}{s_{j},Y},\;\underset{k=2}{\underset{︸}{s_{i},T,a_{T}}}\right)\cdot P_{Disease}^{k=2}\left(s_{j},Y,s_{i},T\right),$$

where the last term on the right-hand side is calculated using the infection trajectories of individuals generated from the simulation. For example, using the full model, we have $P_{Disease}^{k=2}\left(s_{j},Y,s_{i},T\right)=\rho_{Disease}^{k=2}\left(s_{j},s_{i}\right)\cdot E\left[\psi_{Disease}^{k=2}\right]$, where $E\left[\psi_{Disease}^{k=2}\right]$ averages over the relative probability of disease by antigenic distance for the cohort of individuals with birth year $T-a_{T}$ who acquire primary infection with serotype $s_{j}$ at year Y and secondary infection with serotype $s_{i}$ at year T in simulation. For the serotype-pair model and the serotype model, the probability of disease from a secondary infection is calculated using Eq. (5) and Eq. (6), respectively.

Finally, in Figures 4 and S6, the changing population disease risk by year and age is calculated by: $\frac{P_{Disease}^{k=2}\left(T,a_{T}\right)}{P_{Infection}^{k=2}\left(T,a_{T}\right)}$.

## Supplementary Material

Supplement 1

## Data Availability

All code and data necessary to reproduce the analyses will be available at the time of publication.
