# Supplementary material for: Antigenic diversity and dengue disease risk
Source: Res Sq. 2023 Aug 2:rs.3.rs-3214507. Preprint. [Version 1] doi: 10.21203/rs.3.rs-3214507/v1 (PMC10418532; doi:10.21203/rs.3.rs-3214507/v1)
Supplement: Supplement 1 [file NIHPPrs3214507v1-supplement-1.pdf]

Supplementary Figures & Tables

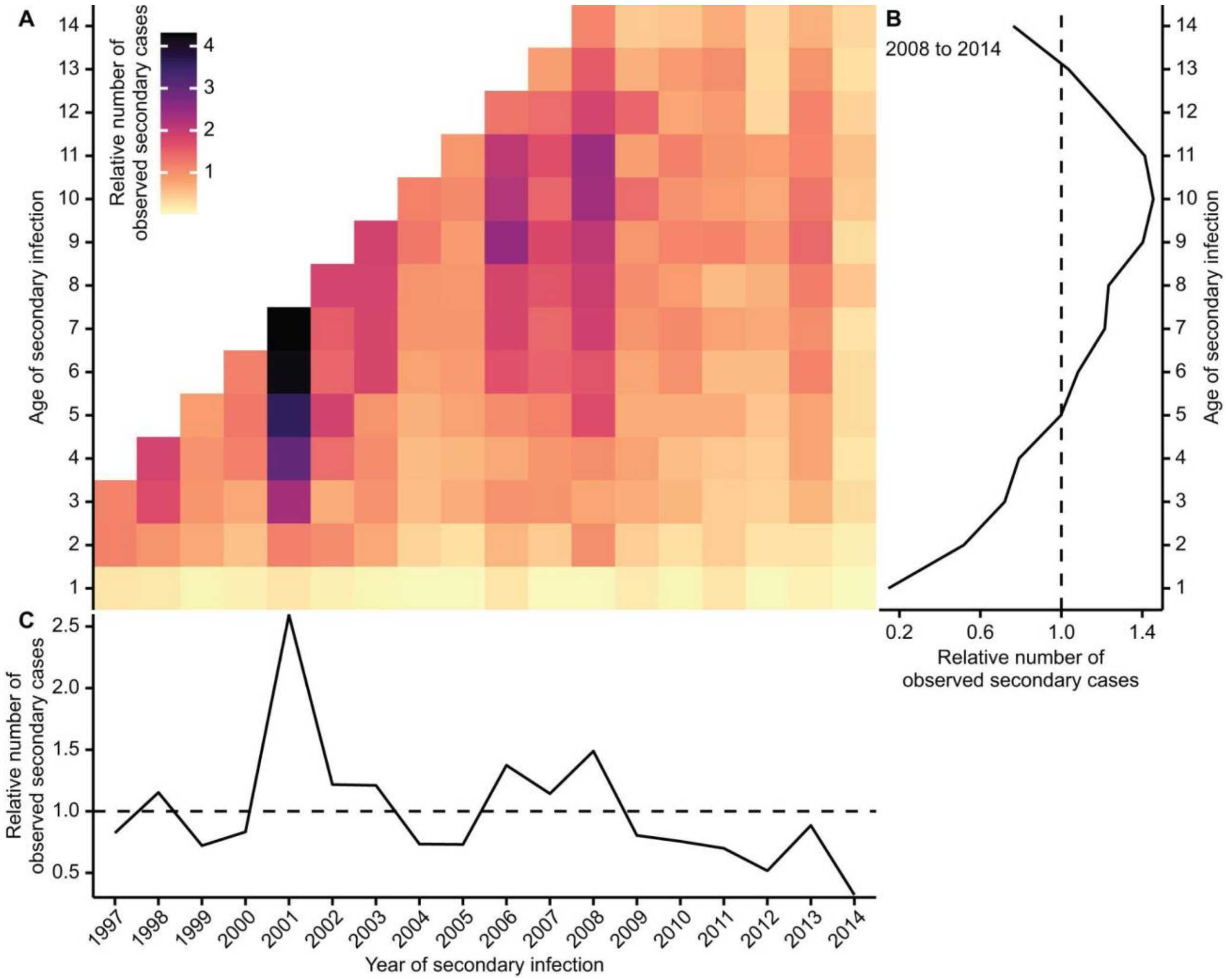

Figure S1. Changing pattern of the secondary dengue cases observed in our surveillance hospital by year and age. (A) The number of hospitalised secondary cases in each year and age, relative to the mean number of hospitalised secondary cases across all years and ages in the study. In each year from 1997 to 2007, older age groups without antigenic distance data were excluded from the analysis (Table S7). Colour in the heat map corresponds to the number of hospitalised secondary cases in each year and age. **(B)** The number of hospitalised secondary cases in each age across years from 2008 to 2014, relative to the mean number of hospitalised secondary cases over all ages across the same years. **(C)** The annual mean number of hospitalised secondary cases that is averaged over different ages in each year, relative to the overall average of the annual mean number of hospitalised secondary cases across all years from 1997 to 2014.

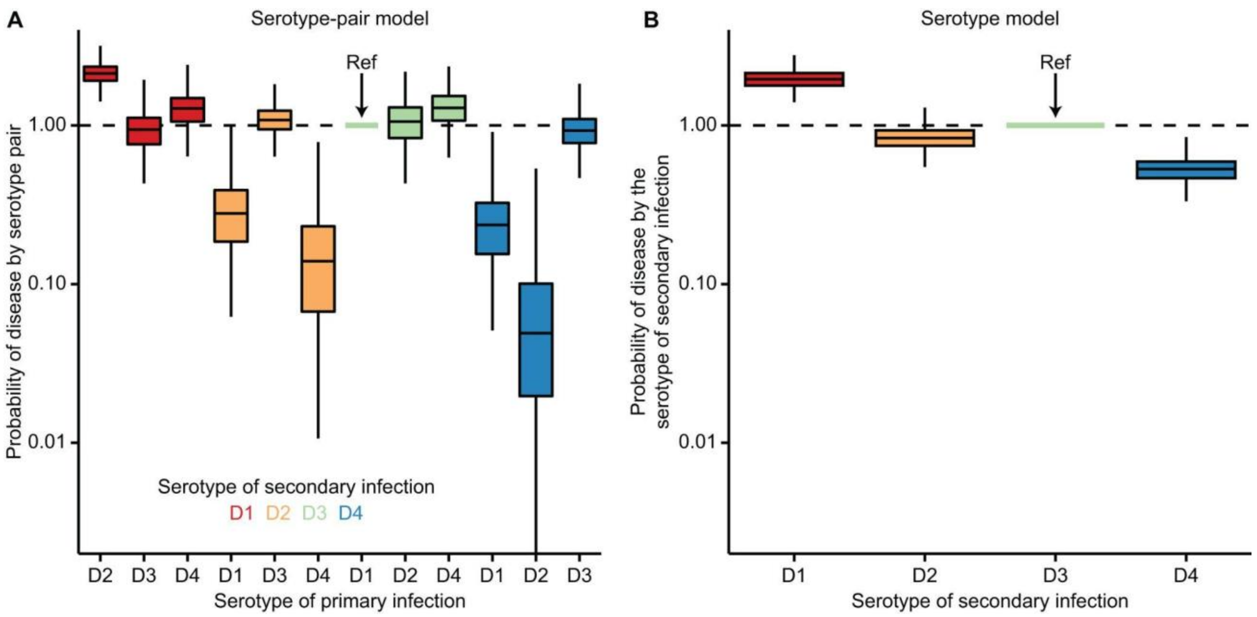

Figure S2. Probability of disease estimated using the model that only considers the serotype-based information. (A) Probability of disease by serotype pair. The inference uses the model where the probability of disease from a secondary infection depends on the serotype of both the primary and secondary infection. Primary DENV-1 followed by secondary DENV-3 serves as the reference for comparison. **(B)** Probability of disease by the serotype of secondary infection. The inference uses the model where the probability of disease from a secondary infection depends on the identity of the secondary infecting serotype only. Secondary DENV-3 serves as the reference for comparison. In each boxplot, the central horizontal line, edges of box, and whiskers indicate the median, interquartile range (IQR), and 1.5 * IQR of the posterior distribution, respectively. Colour indicates the identity of the secondary infecting serotype.

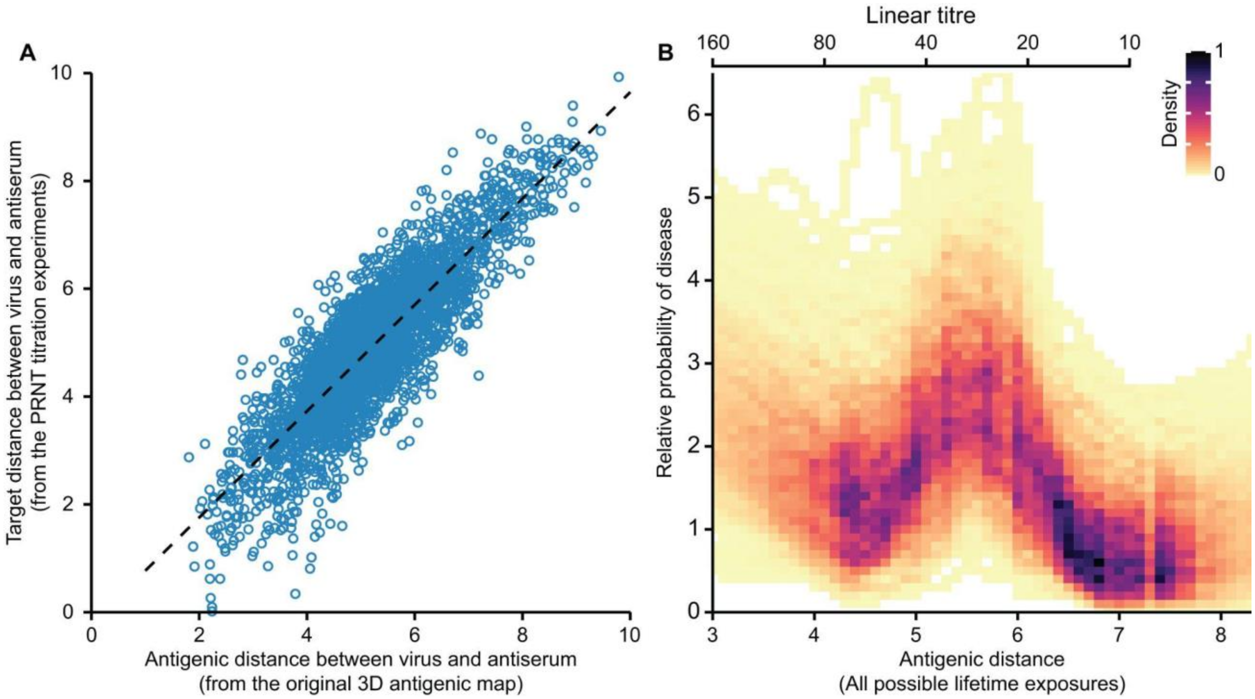

Figure S3. Relationship between the relative probability of disease and the PRNT titre that approximates the measurement using antisera raised against the primary infecting virus to neutralise each secondary infecting virus. (A) Scatter plot compares the antigenic distance derived from the original 3D antigenic map and the target distance derived from the PRNT titration experiments for each pair of virus and antiserum[40]. This analysis excludes all the pairwise virus and antiserum that are within the same serotype, with a titre below limit of detection (e.g., <20, 'threshold'), or with no titre measurement. With the remaining 3,312 data points of titres, linear regression suggests the relationship between the target distance and the antigenic distance as: $\text{target distance} \, \square \, 0.986 * \text{antigenic distance} - 0.216$. With the ensuing target distance, the absolute titre is approximated by $2^{\left(\overline{b_j} - \text{target distance}\right)}$, where $\overline{b_j}$ = 10.11 is set to the overall median of column basis[13,40] across all antisera, with each column basis j being the *log2* of the maximum titre for antiserum j. **(B)** Analogous to Figure 2B but with the top axis showing the absolute PRNT titre translated from the antigenic distance using the approximation derived from (A).

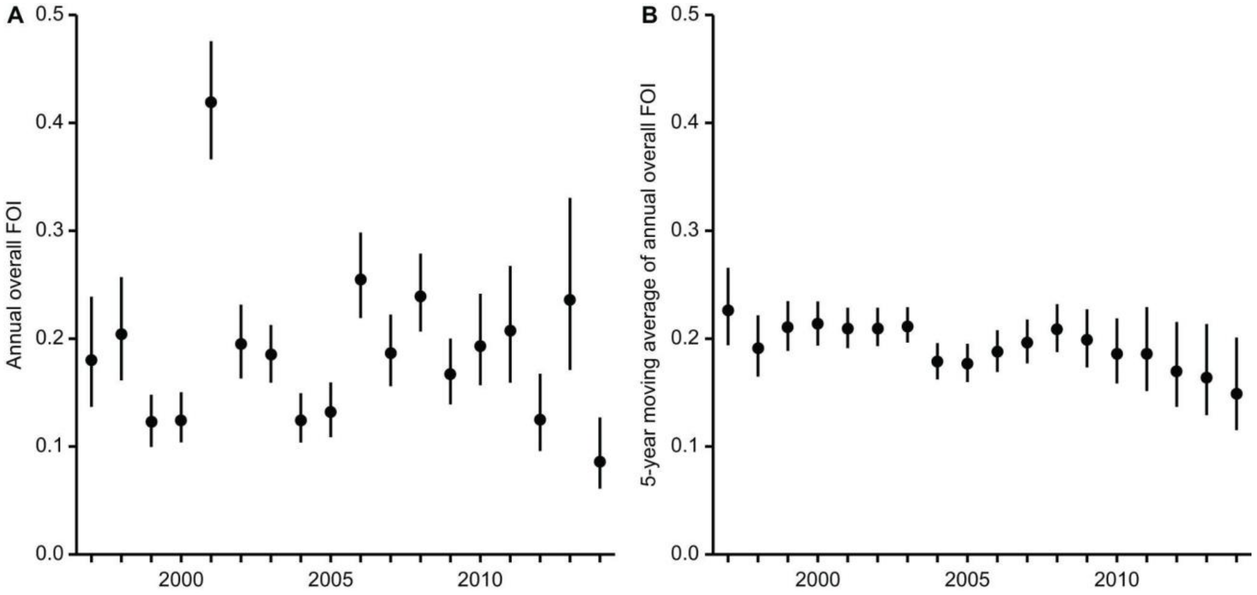

Figure S4. Estimated overall force of infection (FOI) across four serotypes per year. (A) Annual overall FOI across four serotypes. **(B)** Five-year moving average of the annual overall FOI. The inference was based on the full model, with the probability of disease from a secondary infection depending not only on the serotype of the primary and secondary infections, but also on the antigenic distance between the two infecting viruses. Dots and error bars indicate the posterior median and 95% CrI.

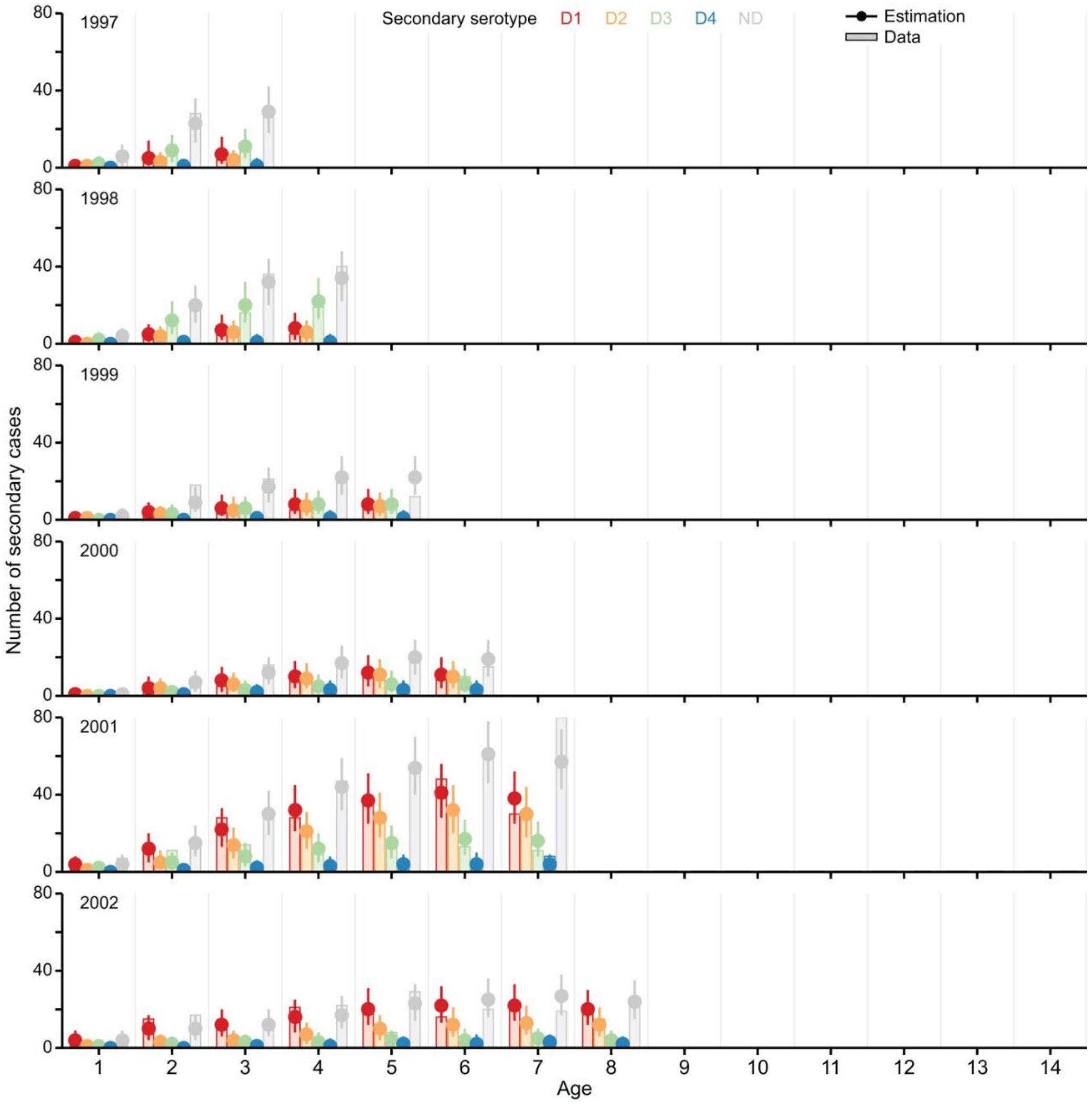

Secondary serotype D1 D2 D3 D4 ND
Estimation
Data
1997
1998
1999
2000
2001
2002
80
40
0
Number of secondary cases
1 2 3 4 5 6 7 8 9 10 11 12 13 14
Age

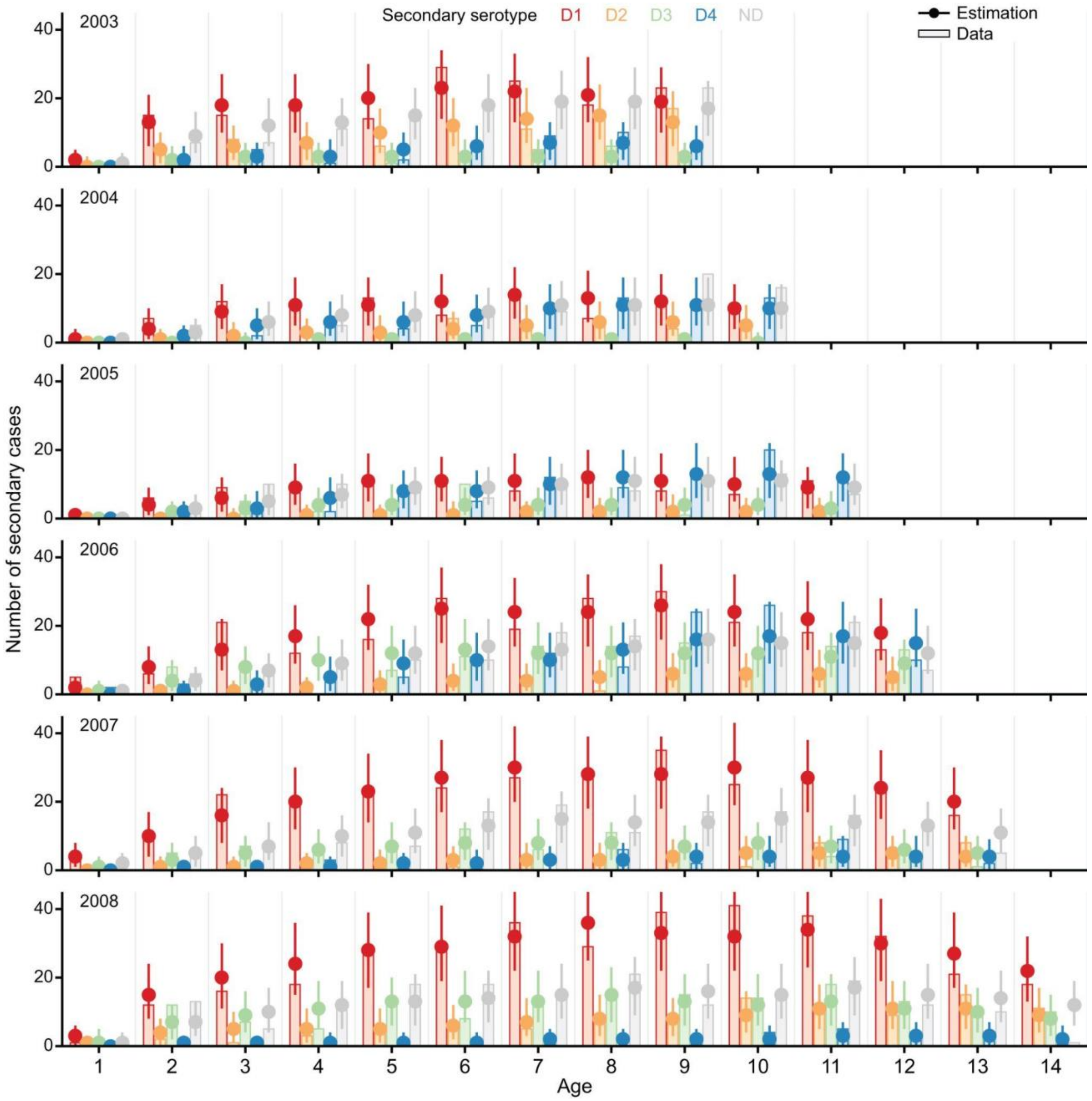
Secondary serotype D1 D2 D3 D4 ND
Estimation
Data
2003
2004
2005
2006
2007
2008
Number of secondary cases
Age
0
20
40
1 2 3 4 5 6 7 8 9 10 11 12 13 14

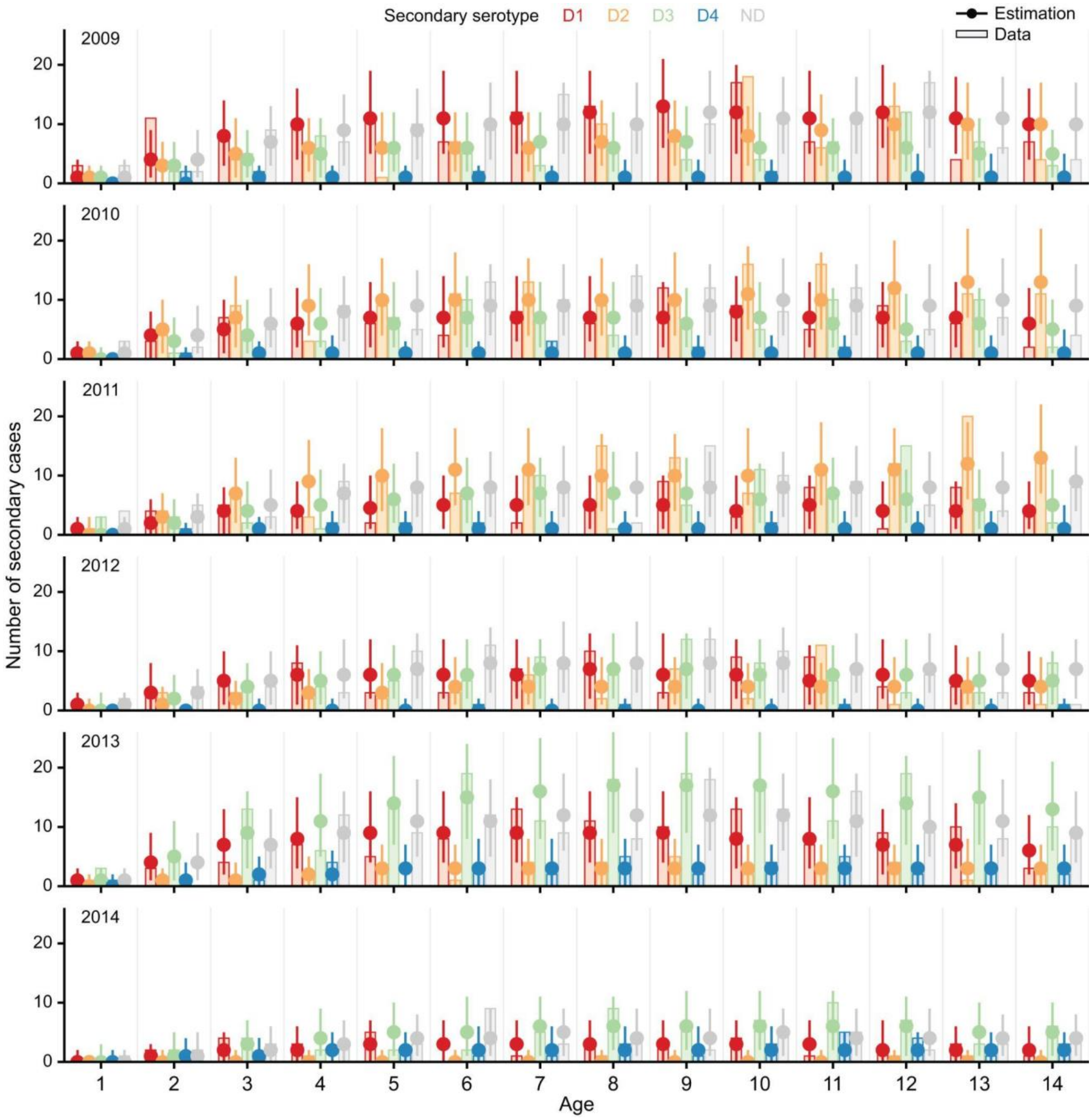

Figure S5. Reconstruction of the yearly hospitalised secondary dengue cases by serotype and age from 1997 to 2014. Vertical bars show the serotype and age-specific counts of secondary cases observed in our surveillance hospital. Dots and error bars indicate the median and 95% CrI of the corresponding case counts estimated by simulating the infection histories of individuals using posteriors inferred from the full model (Methods). In each year from 1997 to 2007, older age groups without antigenic distance data were excluded from the analysis (Table S7). The observed and estimated case counts are coloured by the identity of the secondary serotype. D1 to D4 indicate the secondary cases of each serotype. ND indicates cases without serotype information.

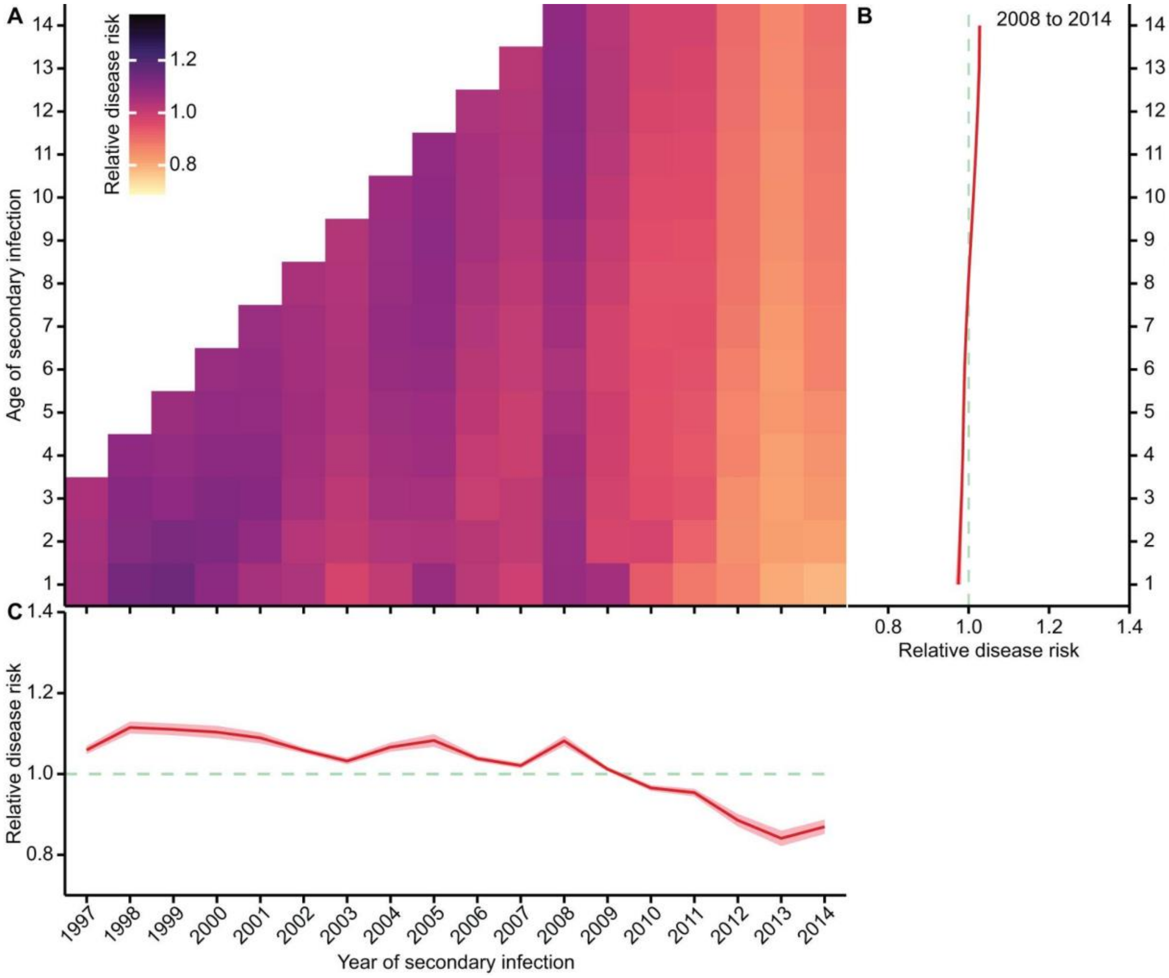

Figure S6. Comparative analysis of the changing population disease risk by year and age. (A) Relative disease risk by year and age with respect to the overall average of disease risks across all years and ages in the study. The estimation averages over 2,000 stochastic simulation realisations of the infection histories of individuals in Bangkok, using 40 randomly selected posteriors from the full model. To compare with Figure 4, given each posterior of parameters, the simulation here assumes that all four serotypes circulate with the same constant force of infection across all years, but the antigenic properties of the circulating viruses remain the same as before. The heat map colour corresponds to the population disease risk (Methods). **(B)** Mean relative disease risk for individuals of each age across years from 2008 to 2014. **(C)** Mean relative disease risk for individuals acquiring secondary infection in each year, with the estimated relative disease risk being averaged over different ages in the same year. In (B) and (C), the line and shaded regions indicate the mean and 95% confidence interval of the estimation.

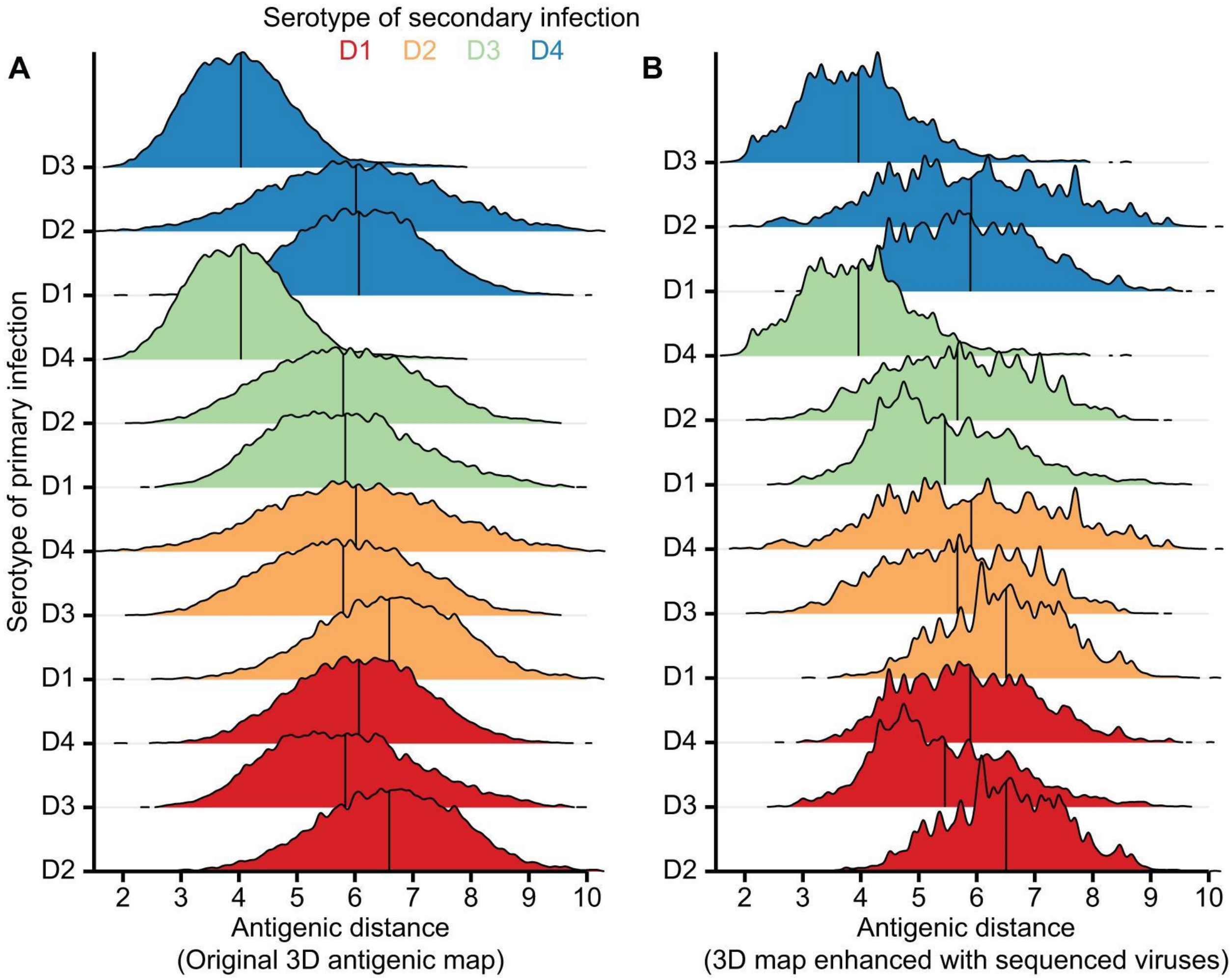

Figure S7. Distribution of antigenic distances separating viruses that are from different serotypes and not isolated in the same year. (A) Using antigenic distance data derived from the original 3D antigenic map. **(B)** Using antigenic distance data derived from the 3D antigenic map enhanced with sequenced viruses. The distributions are stratified by the serotype pair, and coloured by the identity of the secondary serotype. Unlike Figure 1D, Figure S7B uses the inter-serotype antigenic distances without considering the order of the possible infecting viruses.

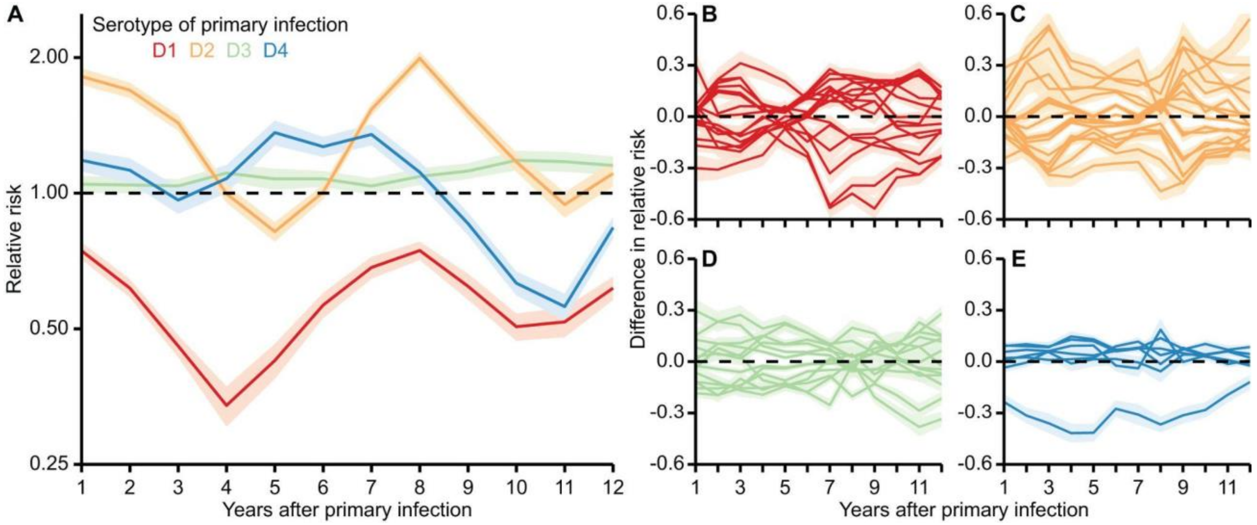

Figure S8. Effects of antigenic imprinting on subsequent disease risk. (A) Disease risk from a secondary infection in each year after primary infection if imprinted by one serotype, relative to the average annual disease risk from a secondary infection if imprinted by any virus. The lines and shaded regions indicate the mean and 95% confidence interval of the disease risk estimated by simulating the infection histories of individuals using posteriors from the full model. Colour indicates the serotype of primary infection. **(B)** Difference in the subsequent disease risk if primed by a given DENV-1 virus as compared to the average disease risk from being primed by any DENV-1 virus. **(C)** to **(E)** Analogous to (B) but for viruses of primary DENV-2 to DENV-4, respectively. The simulation takes individuals from a single birth cohort of 1998, with all of their primary infections assumed to occur in 2000.

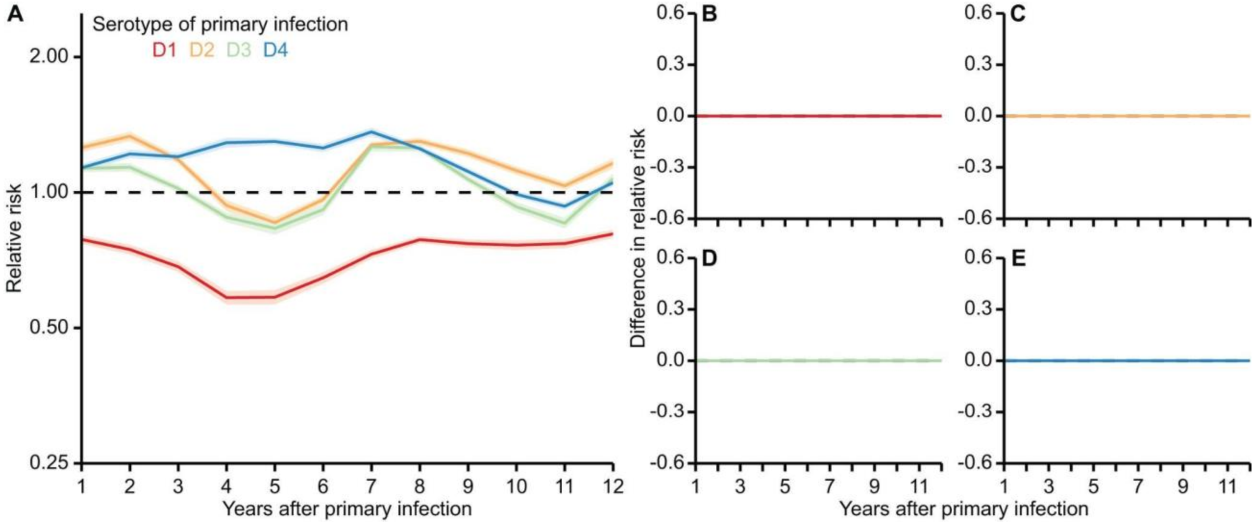

Figure S9. Analogous to Figure S8 but with disease risk estimated by simulating the infection histories of individuals using posteriors from the serotype alone model (i.e., the probability of disease from a secondary infection depending on the serotype of the secondary infection only).

Table S1. Comparison of three models. The serotype model assumes that the probability of disease given a secondary infection only depends on the identity of the secondary infecting serotype. The serotype-pair model assumes that the probability of disease given a secondary infection depends on the serotype of both the primary and secondary infection. The full model assumes that the probability of disease given a secondary infection depends not only on the serotype of the primary and secondary infection, but also on the antigenic distance between the two viruses that are responsible for the primary and secondary infections.

Model	Leave-one-out (LOO) cross-validation [49,50]		Deviance information criterion (DIC) [51]	
	LOOIC [a] (95% CI [d])	p_loo [b] (95% CI [d])	DIC	p_D [c]
Serotype model	4219.7 (4098.8–4340.6)	72.7 (65.4–80.0)	4219.9	80.7
Serotype-pair model	4157.0 (4037.0–4277.0)	76.4 (68.8–84.0)	4156.6	84.8
Full model (L = 10) [e]	4158.3 (4039.1–4277.5)	78.5 (70.9–86.1)	4125.0	54.6

[a] LOO information criterion (LOOIC)[50].

[b] The effective number of parameters (p_loo) calculated using the LOO method[50].

[c] The effective number of parameters (p_D) estimated using the posterior median of parameters[52].

[d] Confidence interval.

[e] The number of knots L used to construct the B-spline basis functions of the antigenic distance in the full model (see Methods).

Table S2. Comparison of the full model by varying the number of knots *L* used to construct the B-spline basis functions. Detailed model structure is provided in the section 'B-spline method' in Methods.

The number of knots L in the full model	Leave-one-out (LOO) cross-validation [49]		Deviance information criterion (DIC) [51]	
	LOOIC [a] (95% CI [d])	p_loo [b] (95% CI [d])	DIC	p_D [c]
$L = 8$	4159.8 (4040.2–4279.4)	78.0 (70.4–85.6)	4139.3	66.6
$L = 10$	4158.3 (4039.1–4277.5)	78.5 (70.9–86.1)	4125.0	54.6
$L = 11$	4157.2 (4038.0–4276.4)	78.4 (70.8–86.0)	4119.1	49.5

[a] LOO information criterion (LOOIC)[50].
[b] The effective number of parameters (p_loo) calculated using the LOO method[50].
[c] The effective number of parameters (p_D) estimated using the posterior median of parameters[52].
[d] Confidence interval.

Table S3. The mean, standard deviation (SD), and coefficient of variation (CV) of the antigenic distances separating viruses that are possibly responsible for an individual's primary and secondary infections. Stratification of the antigenic distances is based on the two possible sequential infecting serotypes.

Secondary serotype	Primary serotype	Distribution of antigenic distances		
		Mean	SD	CV
1	2	6.56	1.02	0.16
1	3	5.32	1.08	0.20
1	4	5.92	1.14	0.19
2	1	6.46	1.04	0.16
2	3	5.54	1.14	0.21
2	4	5.77	1.52	0.26
3	1	5.58	1.34	0.24
3	2	5.86	1.41	0.24
3	4	4.17	0.97	0.23
4	1	5.68	1.15	0.20
4	2	5.82	1.50	0.26
4	3	3.82	0.98	0.26

Table S4. Mean period in years between the peaks of the annual force of infection of each serotype. The full model was used to estimate parameters. The posterior median and 95% CrI were estimated using 1,200 posterior samples obtained after the thinning of MCMC chains (Methods).

	Mean period in years
DENV-1	7 (95% CrI: 6-7)
DENV-2	6 (95% CrI: 5-11)
DENV-3	6 (95% CrI: 4.75-7)
DENV-4	6 (95% CrI: 4.75-8)

Table S5. Spearman's correlation coefficient between the annual force of infection of the different serotypes. The full model was used to estimate parameters. The posterior median and 95% CrI were estimated using 1,200 posterior samples obtained after the thinning of MCMC chains (Methods).

		Spearman's correlation coefficient
DENV-1	DENV-2	0.23 (95% CrI: 0.08, 0.41)
DENV-1	DENV-3	0.17 (95% CrI: -0.07, 0.42)
DENV-1	DENV-4	0.09 (95% CrI: -0.19, 0.37)
DENV-2	DENV-3	0.01 (95% CrI: -0.18, 0.23)
DENV-2	DENV-4	-0.19 (95% CrI: -0.43, 0.01)
DENV-3	DENV-4	-0.31 (95% CrI: -0.49, -0.08)

Table S6. Spearman's correlation coefficient between the annual force of infection and the mean number of hospitalised cases across all ages for each serotype in each year. The full model was used to estimate parameters. The posterior median and 95% CrI were estimated using 1,200 posterior samples obtained after the thinning of MCMC chains (Methods).

	Spearman's correlation coefficient
DENV-1	0.80 (95% CrI: 0.68-0.92)
DENV-2	0.84 (95% CrI: 0.74-0.92)
DENV-3	0.80 (95% CrI: 0.71-0.89)
DENV-4	0.85 (95% CrI: 0.72-0.94)

Table S7. Age groups analysed per year.

	Age	
1997	1	3
1998	1	4
1999	1	5
2000	1	6
2001	1	7
2002	1	8
2003	1	9
2004	1	10
2005	1	11
2006	1	12
2007	1	13
2008	1	14
2009	1	14
2010	1	14
2011	1	14
2012	1	14
2013	1	14
2014	1	14
